# Germline protein, Cup, non-cell autonomously limits migratory cell fate in *Drosophila* oogenesis

**DOI:** 10.1371/journal.pgen.1010631

**Published:** 2023-02-15

**Authors:** Banhisikha Saha, Sayan Acharjee, Gaurab Ghosh, Purbasa Dasgupta, Mohit Prasad

**Affiliations:** 1 Department of Biological Sciences Indian Institute of Science Education & Research- Kolkata Mohanpur Campus Mohanpur, Nadia, West Bengal, India; 2 Laboratory of Malaria and Vector Research, National Institute of Allergy and Infectious Diseases, NIH, Rockville, Maryland, United States of America; HudsonAlpha Institute for Biotechnology, UNITED STATES

## Abstract

Specification of migratory cell fate from a stationary population is complex and indispensable both for metazoan development as well for the progression of the pathological condition like tumor metastasis. Though this cell fate transformation is widely prevalent, the molecular understanding of this phenomenon remains largely elusive. We have employed the model of border cells (BC) in *Drosophila* oogenesis and identified germline activity of an RNA binding protein, Cup that limits acquisition of migratory cell fate from the neighbouring follicle epithelial cells. As activation of JAK-STAT in the follicle cells is critical for BC specification, our data suggest that Cup, non-cell autonomously restricts the domain of JAK-STAT by activating Notch in the follicle cells. Employing genetics and Delta endocytosis assay, we demonstrate that Cup regulates Delta recycling in the nurse cells through Rab11GTPase thus facilitating Notch activation in the adjacent follicle cells. Since Notch and JAK-STAT are antagonistic, we propose that germline Cup functions through Notch and JAK-STAT to modulate BC fate specification from their static epithelial progenitors.

## Author summary

Transformation of stationary epithelial cells into a migratory fate is critical for development, tissue repair and progression of diseases like tumor metastasis. We propose a novel role for the germline soma communication in modulating migratory border cell fate specification from the somatic follicle cells using the *Drosophila* oogenesis model. By coupling fly genetics, immunohistochemistry and live endocytosis, we propose that germline protein, Cup, modulates Delta recycling thus potentiating Notch activation and fine tuning STAT stimulation in the adjacent somatic follicle cells. A fine balance between the Notch and STAT signalling aids in specifying an optimum number of follicle cells to acquire migratory border cell fate.

## Introduction

Transformation of a stationary epithelial cell population into a migratory one is critical not simply for normal metazoan development but is also linked to various pathological conditions including tumor cell metastasis. [1–3]. In fact, the inappropriate acquisition of migratory capabilities by the cells from solid tumors underlies the high degree of fatality associated with metastasis [4–7]. Cells acquire migratory potential via diverse mechanisms which can be broadly classified into two categories: autonomous and regulative or nonautonomous [8,9]. Regulative or nonautonomous communication is more prevalent during such type of cell fate transformation as metazoans employ diverse modes of cell-cell communication.

Border cells (BCs) in *Drosophila* oogenesis have emerged as an excellent genetic model system for studying how stationary epithelial cells transition into motile cells [10]. *Drosophila* oogenesis is a synchronized developmental process consisting of 14 stages of interconnected oval egg chambers [11,12]. Each egg chamber consists of 16 central germline cells, of which only a single cell acquires the oocyte identity, while the remaining 15 cells become nurse cells involved in nourishing the growing oocyte [13–15]. Enveloping the germline cells is a single layer of approximately 750 follicular epithelial cells. A pair of specialized follicle cells, called the polar cells, mark both the anterior and posterior ends of the egg chamber [16] During the germarium stage, the activity of Delta ligand emanating from the nurse cells activates Notch signaling which allows specification of the anterior polar cells [17]. Subsequently, the second round of Delta mediated Notch activation inhibits the proliferation of the follicle cells in stage 6–7 egg chambers and assists their differentiation into distinct cell fates [18,19]. The polar cells secrete cytokine, Unpaired (Upd) which activates the JAK-STAT pathway to confer migratory fate onto a select group of 4–6 anterior follicle cells (AFCs) [20,21] also termed border cells (BCs). BCs undergo partial epithelial to mesenchymal fate transition and initiate posterior movement towards the oocyte [14]. The BCs are marked by the STAT-mediated activation of CEBP transcription factor, Slow border cells (Slbo) [21–23]. After the BCs are specified their posterior movement is guided under the influence of combined yet graded action of growth factors (PVF1-Platelet Derived Growth Factor and Vascular Endothelial Growth Factor-related Factor 1 and EGF-Epidermal growth factor) secreted from the oocyte [24–26]. After the BC cluster reaches the oocyte, it aids in the formation of a channel in the micropyle. This channel permits sperm entry during fertilization [23]. Any defect in BC specification or their efficient movement, impedes micropyle function, rendering eggs sterile.

The JAK-STAT signaling in the AFCs is carefully modulated at multiple levels to recruit an optimum number of FCs to BCs fate (generally 4–6 cells). First, both the production and the distribution of Upd ligand are regulated to form a gradient across the anterior follicle cells. Second, Yorkie, a component of the Hippo signaling, negatively regulates Upd production from the polar cells [27]. Furthermore, the Glypicans, Dally, and Dally-like shape the distribution of Upd ligand, thus calibrating STAT activation and BC fate specification [28]. Within the AFCs, various intracellular components modulate the STAT activity. The suppressor of Cytokine Signaling (SOCS36E) regulates the ubiquitination of several components of the JAK-STAT pathway to limit STAT activation [29,30]. In addition, other checkpoints operate at the level of transcription. In the follicle cells (FCs), antagonistic interactions between STAT and transcriptional repressor Apontic, restrict the domain of STAT activation, thereby limiting BC fate [31]. A recent study shows that Insulin signaling also constrains BC fate by stabilizing the negative regulator SOCS36E in the AFCs [32]. Thus, the JAK-STAT pathway is regulated at multiple levels in the somatic FCs to give rise to a fixed number of BCs during *Drosophila* oogenesis. Since interaction between germline nurse cells and somatic FCs is critical for oogenesis progression and polar cell fate specification, we were curious to examine if the germline cells also have any direct role in BC fate specification [33].

In this study, we report a novel role of nurse cells in BC fate specification. Specifically, our data suggest that germline-specific activity of Cup, non-cell autonomously modulates Notch signaling in the AFCs. As Notch and JAK-STAT signaling work in an antagonistic fashion, Cup mutants exhibit an excess number of BCs likely due to elevated STAT in the AFCs. Further, we demonstrate that Cup mutants exhibit aberrant actin cytoskeleton and enrichment of Delta puncta in the nurse cell cytoplasm. Employing classical genetics and tissue immunohistochemistry in various genetic backgrounds, we show that Cup maintains the integrity of the germline cytoskeleton and modulates Delta trafficking in the nurse cells. Also, overexpression of constitutively active Rab11GTPase in the germline of *cup* mutants can rescue the excessive BC fate. Together these data argue that recycling Delta in the germline nurse cells is critical for Notch activation in the AFCs of vitellogenic egg chambers. Activation of Notch in the AFCs modulates STAT activity, thus controlling the total number of AFCs that acquire BC fate.

## Results

### The germline specific function of cup affects the size of the somatic BC cluster

In *Drosophila melanogaster* both proper specification and migration of BCs are critical determinants of female fertility. Several autocrine, and paracrine factors associated with AFCs mediate the specification of BCs. Though signals from the nurse cells regulate the polar and stalk cell fate in previtellogenic egg chambers (stages 1–2), it is not clear if the germline can directly impact the specification of BCs [34]. Hence, we sought to assess if the germline nurse cells directly participate in the specification of somatic BCs during early vitellogenesis.

**Fig 1:**
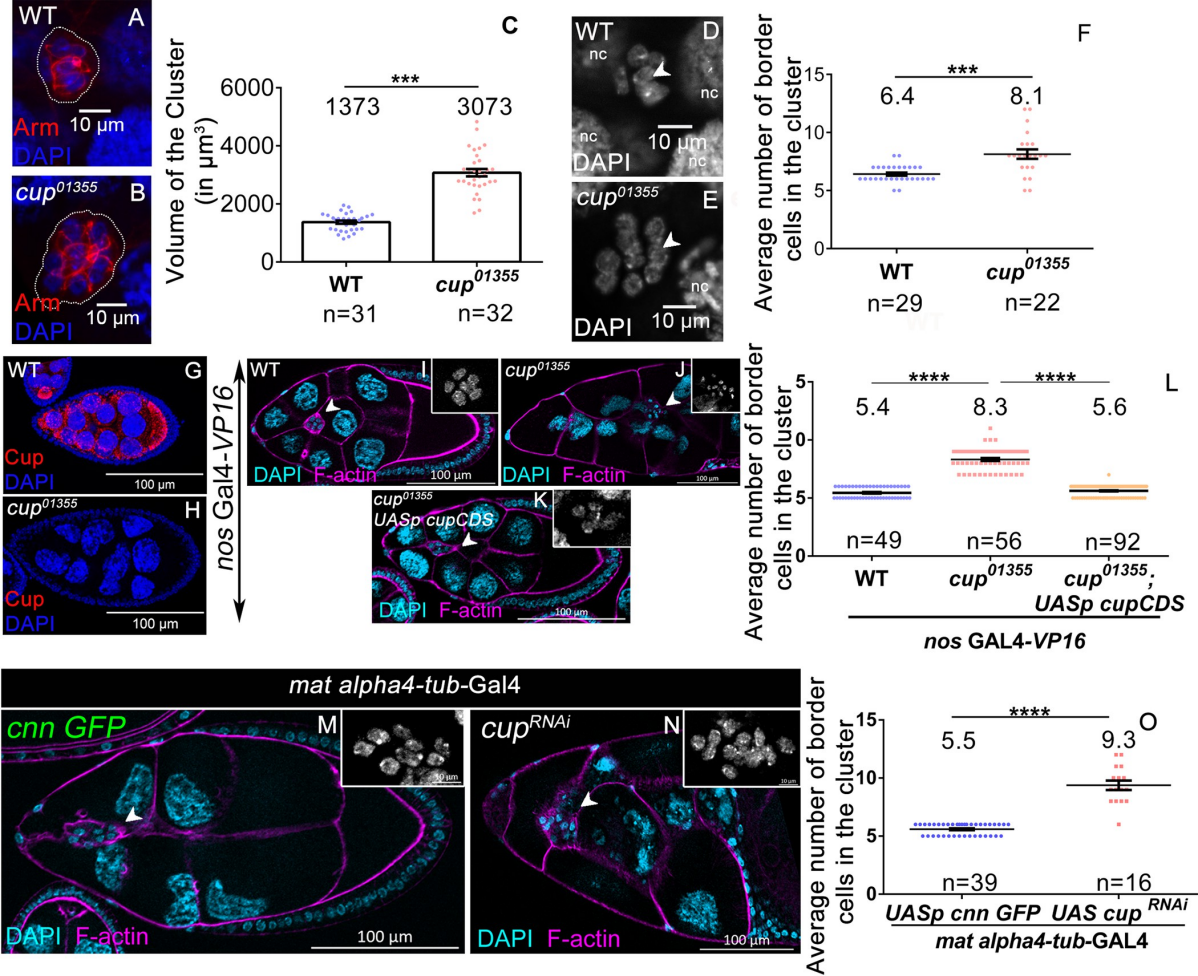
Cup functions in the germline cells to limit the size of the BC cluster. (**A-C**) *cup*^*01355*^ egg chambers exhibit larger border cell cluster, Armadillo (red), DAPI (blue), White dotted line marks BC cluster. (**D-F**) *cup*^*01355*^ egg chambers exhibit increased cell count, DAPI (grey), compared to wild type. (**G-H**) *cup*^*01355*^ egg chambers lack Cup Expression, Cup (red), DAPI (blue). (**I-L**) Increased BC number (white arrow) is rescued by *UASp-CupCDS*, driven by *nos*. GAL4*-VP16* in *cup*^*01355*^ egg chambers, F-actin (magenta), DAPI (cyan, grey in inset). Student t-test, p value between WT and *cup*^*01355*^ is <0.0001 indicated by ****, p value between *cup*^*01355*^ and *UASp-CupCDS* in *cup*^*01355*^ background is <0.0001 indicated by ****, p value between WT and *UASp-CupCDS* in *cup*^*01355*^ background is 0.0698 indicated by ns (not significant). **(M-O)** Down regulation of Cup in germline by overexpressing *cup*^*RNAi*^ with germline *mat alpha4-tub-*GAL4 exhibit increased border cell count compared with control, F-actin (magenta), DAPI (cyan, inset in grey). n represents the number of egg chambers evaluated. SEM represent the error bars.

To address this question, we narrowed down 14 candidate genes known to be expressed in the nurse cells. We also ensured that mutations in these genes result in female sterility (S1 Table). Among these 14 genes, we examined the status of BC fate specification in 3 mutant lines that are homozygous viable. We reasoned that the size of BC clusters in the homozygous mutant egg chambers will be altered if the gene product directly controls BC specification. We measured the size of the BC cluster for each of the three homozygous mutant lines and found that mutation in the Cup gene (*cup*^*01355*^) resulted in the largest BC cluster among the three mutants (3073.40±127.59μm^3^ SEM, n = 32 clusters) compared to the WT (1373.33±54.86 μm^3^ SEM, n = 31 clusters) (Fig 1A–1C). The BC clusters of *Gprk*^*06923*^ and *cdc27*^*L7123*^ mutants were also larger than the WT (*Gprk*^*06923*^: 2342±98.6 μm^3^ SEM n = 31; *cdc27*^*L7123*^: 1926±121.4 μm^3^ SEM, n = 29) (S1A–S1D Fig).

Cup protein has been shown to regulate the translation and stability of several maternal mRNAs including *oskar* and *nanos* during *Drosophila* oogenesis [35–37]. *cup*^*01355*^, a hypomorphic allele, has a *P-lacZ* insertion in the untranslated region of the first exon of the *cup* gene. It belongs to the least severe class of alleles where the phenotype manifests only during post-vitellogenic stages of *Drosophila* oogenesis [38].

To establish that the larger clusters observed in *cup*^*01355*^ homozygotes were indeed due to the altered number of BCs, we stained the egg chambers with DAPI to quantify the number of BCs. Consistent with our expectation, we observed that the number of BCs in *cup*^*01355*^ mutant egg chambers was higher (8.13±0.4 SEM, n = 22) compared to WT (6.41±0.13 SEM, n = 29) (Fig 1D–1F). To exclude the possibility that a second-site mutation induced the phenotype, we analyzed BCs numbers in the heterozygous genetic background of *cup*^*01355*^ and *cup*^*8*^ background. *cup*^*8*^ is an ethyl methyl sulfonate induced allele that was independently isolated and thus has a different genetic background. It exhibits morphological defects in stage 8–9 egg chambers [39]. Satisfyingly, we also observed an increase in the number of BCs compared to the control in *cup*^*8*^*/ cup*^*01355*^ trans-heterozygous background (*cup*^*8*^*/cup*^*01355*^-8.37±0.11 SEM, wild type-5.34±0.07 SEM, n≥50 egg chambers) (S1E–S1G Fig). Altogether these data suggest that Cup modulates the number of BCs in the migrating clusters of the developing egg chambers. Next, we sought to examine if Cup activity is required in the germline cells or somatic follicular cells to influence BC fate. We stained the egg chambers with anti-Cup antibodies and observed that Cup is highly expressed in the cytoplasm of the germline nurse cells both in the early and late stages of oogenesis (Fig 1G). Consistent with previously published reports, we failed to detect any Cup protein in the somatic FCs (Fig 1G) [38]. Since *cup*^*01355*^ is a hypomorphic allele, we examined the levels of *cup* transcript and protein in *cup*^*01355*^ ovaries. We observed reduced levels of *cup* transcript (1/10th of WT), and also failed to detect any Cup protein in the homozygous *cup*^*01355*^ egg chambers (Fig 1G–1H, S1H–S1I Fig). The expression analysis of the Cup gene product in WT and *cup*^*01355*^ egg chambers suggested that Cup is primarily expressed in the germline and likely works in a non-cell autonomous manner to specify BC fate. To confirm that Cup activity is not required in the FCs, we employed Mosaic Analysis with a Repressible Cell Marker (MARCM) technique to generate homozygous mutant Cup FCs using a stronger allele of Cup (*cup*^*15*^). *cup*^*15*^ is an EMS allele, and mutant ovaries are known to exhibit a negligible amount of Cup protein as compared to WT [38]. We examined the status of BC fate specification when follicle cells were nearly devoid of Cup protein [40]. As expected, we didn’t observe any significant difference in BC numbers specified in *cup*^*15*^ mutant AFCs (5.68±0.06 SEM, n = 97) compared to WT AFCs (5.76±0.07 SEM, n = 50) (S1J and S1K Fig). To validate that the increased BC number is indeed due to the absence of Cup in the nurse cells, we restored the *cup* activity by expressing the Cup-coding region (Cup-CDS) in *cup*^*01355*^ nurse cells using *mat alpha4-tub-*GAL4. Upon reconstitution of Cup-CDS in *cup*^*01355*^ nurse cells, the BC number was significantly restored close to that of the WT (*cup*^*01355*^-8.32±0.12SEM, rescue-5.62±0.05 SEM, wild type-5.45±0.76 SEM, n≥49 egg chambers (Fig 1I–1L). However, overexpression of Cup-CDS in the anterior follicle cells failed to rescue the elevated number of BCs observed in the *cup* mutant. *(cup*^*01355*^-8.15±0.12SEM, *c306; cup*^*01355*^*; Cup-CDS*-8.02±0.08 SEM, wild type-5.46±0.07 SEM, n≥35 egg chambers) (S1L–S1O Fig)

To further support the non-autonomous role of Cup in border cell fate specification, we downregulated *cup* function in germline nurse cells employing *mat alpha-tub-GAL4* and *cup RNAi*. Since the *mat alpha4-tub-*GAL4 driver has weak expression in the nurse cells of early-stage chambers, it enabled us to evaluate *cup* function during mid-oogenesis. Consistent with our expectation, we observed a higher number of BCs in the migratory cluster compared to the control supporting our conclusion that germline Cup non-cell autonomously affects the BC fate in the AFCs *(Cup*^*RNAi*^-9.375±0.4 SEM n = 16 egg chambers, control-5.59±0.07 SEM, n = 39 egg chambers) (Fig 1M–1O)

Altogether our results suggest that nurse cell specific activity of Cup protein non-cell autonomously modulates the size of the BC cluster which is specified from the overlying somatic AFCs.

### Cup controls BC fate by negatively regulating the JAK-STAT pathway

**Fig 2:**
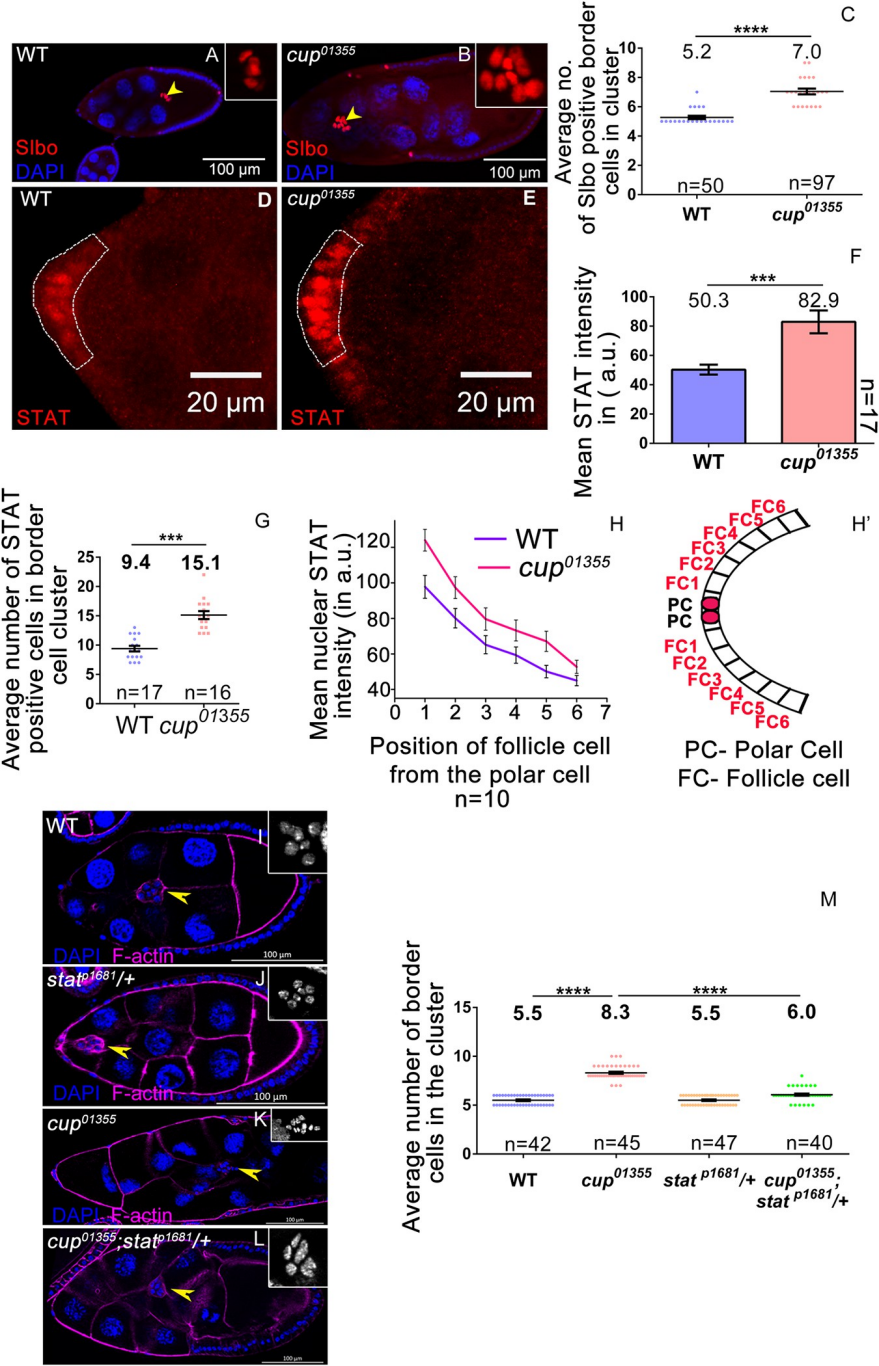
Cup modulates STAT in the follicle cells to limit BC fate. (**A-C**) *cup*^*01355*^ egg chambers exhibit increased Slbo positive cells (yellow arrow head) compared to wild type, Slbo (red), DAPI (blue). (**D-G**) *cup*^*01355*^ egg chambers exhibit higher STAT levels and more STAT positive cells in anterior end of egg chamber as compared to wild type (dotted area), STAT (red). (**H-H’**) STAT level is higher in 6^th^ FC from polar cell in *cup*^*01355*^ egg chambers as compared to control. (**I-M**) Higher BC numbers is rescued when *stat*^*p1681*^*/+* background is introduced in *cup*^*01355*^ homozygous background (yellow arrow heads mark BC), F-actin (magenta), DAPI (blue, grey in inset). Student t-test, p value between WT and *cup*^*01355*^ is <0.0001 indicated by ****, p value between *cup*^*01355*^ and *cup*^*01355*^*; stat*^*p1681*^*/+* is <0.0001 indicated by ****, p value between WT and *cup*^*01355*^*; stat*^*p1681*^*/+* is <0.0001 indicated by ****. n represents the number of egg chambers evaluated. SEM represent the error bars.

Since *cup*^*01355*^ mutant egg chambers exhibit more nuclei in the migrating cluster, we investigated if the extra cells were indeed BCs. To check this, we stained the egg chambers for the Slbo protein which marks the BCs. We observed a significantly higher number of Slbo-positive cells in the cluster (7.04±0.19 SEM, n = 23) of *cup*^*01355*^ egg compared to the WT (5.27±0.11SEM, n = 22) (Fig 2A–2C). This suggests that Cup depletion results in the aberrant specification of BCs from the follicular epithelium. As Notch signaling modulates the mitotic to endocycle switch in developing eggs, we curious to know if an increase in BC numbers in *cup*^*01355*^ background is due to a prolonged mitotic phase [18]. To check this, we compared the expression pattern of two markers associated with proliferating follicle cells, Cut and phospho-Histone 3 (pH3) [18,34]. We examined 170 egg chambers each of WT, and *cup*^*01355*^ and observed no difference in the staining pattern for Cut (S2A–S2H Fig); nor did we observe any pH3-positive cells in stage 8, or higher stage egg chambers in 168 samples analyzed each for WT and *cup*^*01355*^ (S2I–S2N” Fig). As the expression pattern of both Cut and pH3 was similar in both the WT and the *cup*^*01355*^ egg chambers, we ruled out the possibility that extra rounds of mitotic division are responsible for the excess BC numbers observed in the *cup* mutants. In addition, we assessed the total number of follicle cell nuclei in stage 8 egg chambers for both wild-type and *cup*^*01355*^ mutants. We counted the number of follicle cell nuclei plane by plane of a confocal z stack to ensure that each nucleus (DAPI) was counted only once. Satisfyingly we didn’t observe any significant difference in the number of follicle cell nuclei between WT and *cup* mutant egg chambers *(cup*^*01355*^-968.6±6.97 SEM n = 10, wild-type-963.6±5.63 SEM n = 10) (S2O–S2Q Fig). This further supported our claim that inappropriate FC proliferation is not responsible for the elevated number of BCs observed in the *cup*^*01355*^ mutant egg chambers.

Since JAK-STAT signaling activates Slbo expression in the AFCs, we next examined if the increase in the number of BCs in *cup*^*01355*^ mutant egg chambers were linked to enhanced STAT function [21,22]. Nuclear STAT is used as a molecular reporter for assessing the status of JAK-STAT signaling [41]. We quantified nuclear STAT and observed higher levels (1.64-fold) of STAT in *cup*^*01355*^ mutant FCs (82.91±7.76 SEM, n = 17 egg chambers) compared to the WT FCs (50.32±3.36 SEM, n = 17) (Fig 2D–2F). We also observed that the number of AFCs exhibiting distinct nuclear STAT in *cup*^*01355*^ egg chambers (15.12±0.67 SEM, n = 16) was higher compared to WT (9.41±0.47 SEM, n = 17) (Fig 2G). In addition, we observed an elevated number of nuclear STAT positive cells which extend as far as 6th FC from the polar cell in *cup*^*01355*^ egg chambers as opposed to the 3 cells observed in the control (Fig 2H and 2H’). Taken together these results suggest that both the levels and spread of STAT activation are enhanced in *cup*^*01355*^ egg chambers. To assess if the elevated STAT was indeed responsible for excess BCs observed in the *cup*^*01355*^ egg chambers, we compared BC clusters in *cup*^*01355*^ egg chambers in WT and STAT heterozygous background (*stat*^*P1681*^/+). BC number in *the cup*^*01355*^ cluster was reduced in STAT heterozygous background as compared to the *cup*^*01355*^ mutants itself (*cup*^*01355*^-8.31±0.07 SEM, *cup*^*01355*^*; stat*^*P1681*^ */*+-6.0±0.1 SEM, wild type-5.50±0.07 SEM, n≥40 egg chambers). Expectedly, the observed rescue of BC fate was partial likely due to heterozygosity for STAT. Collectively these data suggest that elevated STAT levels are responsible for the enhancement in BC fate specification observed in the *cup*^*01355*^ egg chambers (Fig 2I–2M).

### Loss of cup reduces Notch signaling and increases STAT activity

**Fig 3:**
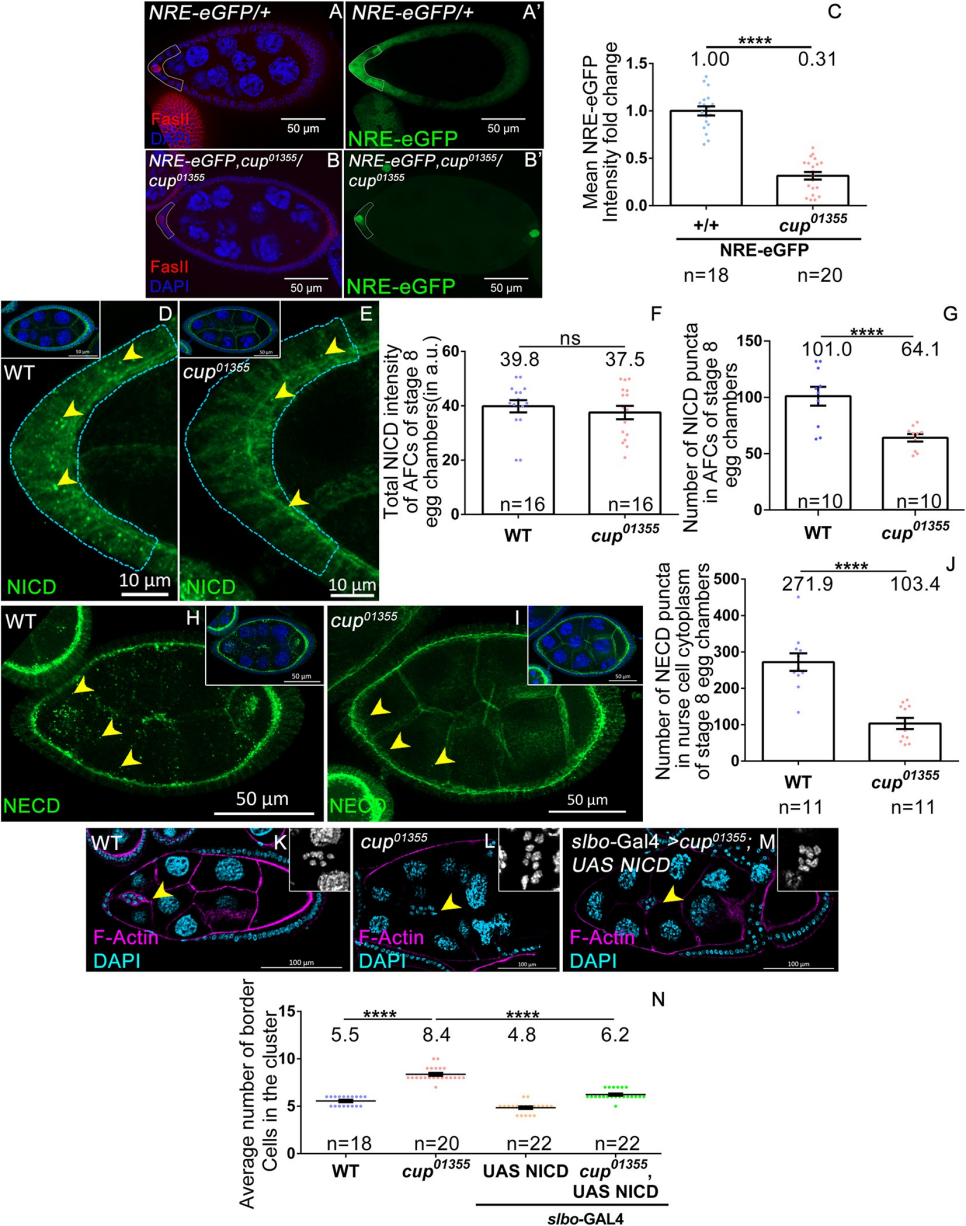
Lower Notch activation in the follicle cells is responsible for the excessive border cell fate observed in the Cup mutants. **(A-C)** Intensity of NRE-eGFP (in green) is significantly decreased in the anterior follicle cells of stage 8 *cup*^*01355*^ egg chambers. **(A-B)** Dotted line marks the anterior follicle cells expressing NRE-eGFP in the indicated genotypes. Depleted FASII (red) staining indicates stage 8 egg chamber. DAPI (blue). **(C)** Quantification of the NRE-eGFP. p value of <0.0001 is indicated by ****, **(D-G)** Stage 8 egg chambers stained with NICD (green). **(D-E)** Reduced number of NICD puncta (yellow arrow head) observed in the anterior follicle cells of *cup*^*01355*^ mutant egg chamber compared to wild type. Inset is the representative egg chambers **(F-G)** Quantification of NICD intensity (F) and puncta (G). Though mean intensity of NICD is not significantly different between *cup*^*01355*^ mutant and wild type follicle cells (F), the number of NICD puncta is significantly reduced (G). (H-J) *cup*^*01355*^ exhibits lesser number of cytoplasmic puncta of NECD (green) (yellow arrow head) in the nurse cell compared to the wild type. (K-N) Increased BC number is rescued by *UAS-NICD*, driven by *slbo-*GAL4 in *cup*^*01355*^ egg chambers, yellow arrow heads mark BC cluster, F-actin (magenta), DAPI (cyan, grey in inset). Student t-test, p value between WT and *cup*^*01355*^ is <0.0001 indicated by ****, p value between *cup*^*01355*^ and *UAS NICD* in *cup*^*01355*^ is <0.0001 indicated by ****, p value between WT and *UAS NICD* in *cup*^*01355*^ is 0.0004 indicated by ***. n represents the number of egg chambers evaluated. SEM represent the error bars.

As higher STAT levels can lead to the specification of an elevated number of BCs, we next decided to analyze why STAT function is higher in *cup*^*01355*^ FCs. JAK-STAT signaling in the AFCs is positively regulated by the Upd ligand produced by the anterior polar cells [20,21]. Since we detected increased JAK-STAT signaling in the Cup mutant FCs, we wondered if this was due to an increase in the number of polar cells. To this end, we examined the pattern of Fasciclin III (FasIII), the lateral membrane protein that marks the junction between the two polar cells [16]. Like the WT, we observed a single distinct junction labeled by FasIII at the anterior and posterior ends of the polar cells in the *cup*^*01355*^ *egg* chambers. This observation suggested that the number of polar cells is unaffected in *cup*^*01355*^ egg chambers (S3A and S3B Fig). We also observed similar FasIII expression in the early stages of oogenesis in both *WT* and *cup*^*01355*^ egg chambers indicating that polar cell fate is unaffected in the *cup*^*01355*^ hypomorphic background (S3C–S3J’ Fig). As the polar cell number is unaffected, we tested if enhanced JAK-STAT signaling was due to transcriptional upregulation of *the upd* gene. To examine this, we measured the expression of *the upd* reporter construct, *upd*-lacZ, and observed no significant difference in the intensity of β-gal antibody staining from *upd*-lacZ between the WT (118.69±7.02 SEM, n = 20) and the *cup*^*01355*^ stage 8 egg chambers (124.73±11.03 SEM, n = 20) (S3K–S3M Fig). Similarly, no change in the β-gal antibody staining intensity was observed in the younger egg chambers between the WT and the *cup*^*01355*^ (S3N–S3V Fig). This ruled out the possibility that elevated *upd* transcription could be responsible for increased STAT signaling in the *cup*^*01355*^ egg chambers (S3K–S3M Fig). This prompted us to explore the possibility of Cup modulating the function of other JAK-STAT regulators which, in turn, may affect BC specification. For instance, the upregulation of STAT activity could be explained by the downregulation of one (or more) of the negative regulators of the JAK-STAT signaling pathway in the *cup*^*01355*^ egg chambers. The known negative regulators of the JAK-STAT signaling include Protein tyrosine phosphatase 61F (Ptp61f), Brahma (Brm), Suppressor of Cytokine Signaling 36E (SOCS36E), and Notch [33,42,43]. Among these, we decided to focus on Notch primarily for two reasons. First, communication between nurse cells and FCs depends on Notch signaling during egg chamber development. Secondly, Notch signaling inhibits JAK-STAT signaling in a context-specific manner in the FCs [33,34]. To assess the level of Notch signaling in the FCs we employed a Notch reporter construct, where the Notch Response Element (NRE) is tagged upstream of eGFP (NRE-eGFP) [44]. NRE consists of binding sites for the transcription factor, Suppressor of Hairless, and the transcriptional activator Grainy head. Activation of Notch signaling leads to the binding of these transcriptional activators to the NRE sequence leading to GFP expression. We checked Notch activity in AFCs by measuring eGFP reporter expression (under NRE) in both control and *cup*^*01355*^ stage 8 egg chambers. Interestingly, we observed a fivefold decrease in the levels of eGFP in the AFCs of *cup*^*01355*^ egg chambers (*cup*^*01355*^-0.2±0.008 SEM, n≥30 egg chambers) compared to the control (1.0±0.06 SEM, n≥30 egg chambers) (Fig 3A–3C). We observed a rescue in the levels of NRE-eGFP when Cup CDS was overexpressed in the nurse cells of *cup*^*01355*^ mutant egg chambers *(cup*^*01355*^-0.4±0.06 SEM n = 10*;cup*^*01355*^*; CupCDS/ nos* GAL4-VP16–1.1±0.3 SEM n = 6, wild type-1.0±0.2 SEM, n = 5 egg chambers) (S4D–S4G Fig). However, over-expression of Cup-CDS in the AFCs failed to rescue the low levels of NRE-eGFP observed in the Cup mutants suggesting that germline-specific function of Cup modulates Notch signaling in the AFCs *(cup*^*01355*^-0.28±0.05 SEM n = 5, *c306* GAL4*; cup*^*01355*^*; CupCDS* -0.26±0.01 SEM n = 12, WT-1.0±0.05 SEM, n = 7 egg chambers) (S4H–S4K Fig). Altogether these data suggest that germline Cup modulates the strength of Notch signaling in the AFCs of developing egg chambers.

To support this observation further, we examined the levels and distribution of Notch in the FC. It is known that ligand binding stimulates two sequential proteolytic cleavages in the Notch receptor generating a fragment with the extracellular domain (NECD) and the other with the intracellular domain (NICD) [45]. The distribution of NICD and NECD is routinely used to evaluate the status of Notch signaling. Ligand stimulation promotes NECD and NICD internalization in the ligand-producing cell and signal-receiving cell respectively [46–48]. We observed numerous NICD and NECD puncta in the WT FC and nurse cells respectively (Fig 3D–3J). The presence of a large number of NICD and NECD puncta suggests that Notch signaling is active in WT FCs. On the contrary, we observed very few internalized puncta of both NICD and NECD in the FC and the nurse cell of the *cup* mutant egg chambers respectively supporting the fact the Notch signaling is downregulated (For NICD; WT-101±8.32 SEM n = 10, *cup*^*01355*^-64.10±3.345 SEM n = 10 (Fig 3G) and for NECD; WT-271.9±24.18 SEM n = 11, *cup*^*01355*^-103.4±15.30 SEM n = 11. (Fig 3J). We observed a similar trend in the distribution of NICD puncta in the *cup*^*8*^*/cup*^*01355*^ background (WT-138.3± 18.9 SEM, n = 10, *cup*^*8*^*/cup*^*01355*^-44.6±7.8 SEM n = 10) (S4A–S4C Fig). However, we didn’t observe any significant difference in the total NICD intensity between the AFCs of WT and the Cup mutant egg chambers (WT-39.8±2.24 SEM n = 16, *cup*^*01355*^-37.5±2.4 SEM n = 10) (Fig 3D–3F) suggesting against the transcriptional downregulation of Notch receptor.

Next we decided to test if simply reducing levels of Notch signaling in *cup*^*01355*^ egg chambers alone can enhance the BC fate specification. Thus, we upregulated Notch signaling in the AFCs of *cup*^*01355*^ egg chambers and examined if this can mitigate the increase in total number of BCs. Notch signaling is required during early as well as late stages of oogenesis. To activate Notch signaling specifically during mid to late stages of oogenesis, we overexpressed the Notch intracellular Domain (UAS-NICD) using the late driver *slbo*-GAL4 [49]. We observed a lower number of BCs in the *cup*^*01355*^ egg chambers overexpressing the NICD *(cup*^*01355*^, UAS-NICD*; slbo-*GAL4- 6.2±0.11 SEM n = 22) compared to the *cup*^*01355*^ mutant egg chambers (*cup*^*01355*^-8.3±0.15 SEM n = 22, WT-5.5±0.1 SEM, n = 18 egg chambers) (Fig 3K–3N). The rescue was partial, possibly due to the late expression of the driver. Taken together these results suggest that the increase in the BC fate observed due to loss of function of *cup* is mediated by the suppression of Notch signaling in the follicle cells. Thus, Cup functions via Notch to modulate the number of AFC that eventually acquire BC fate in the developing egg chambers.

### Cup regulates the nurse cell organization and Delta trafficking

**Fig 4:**
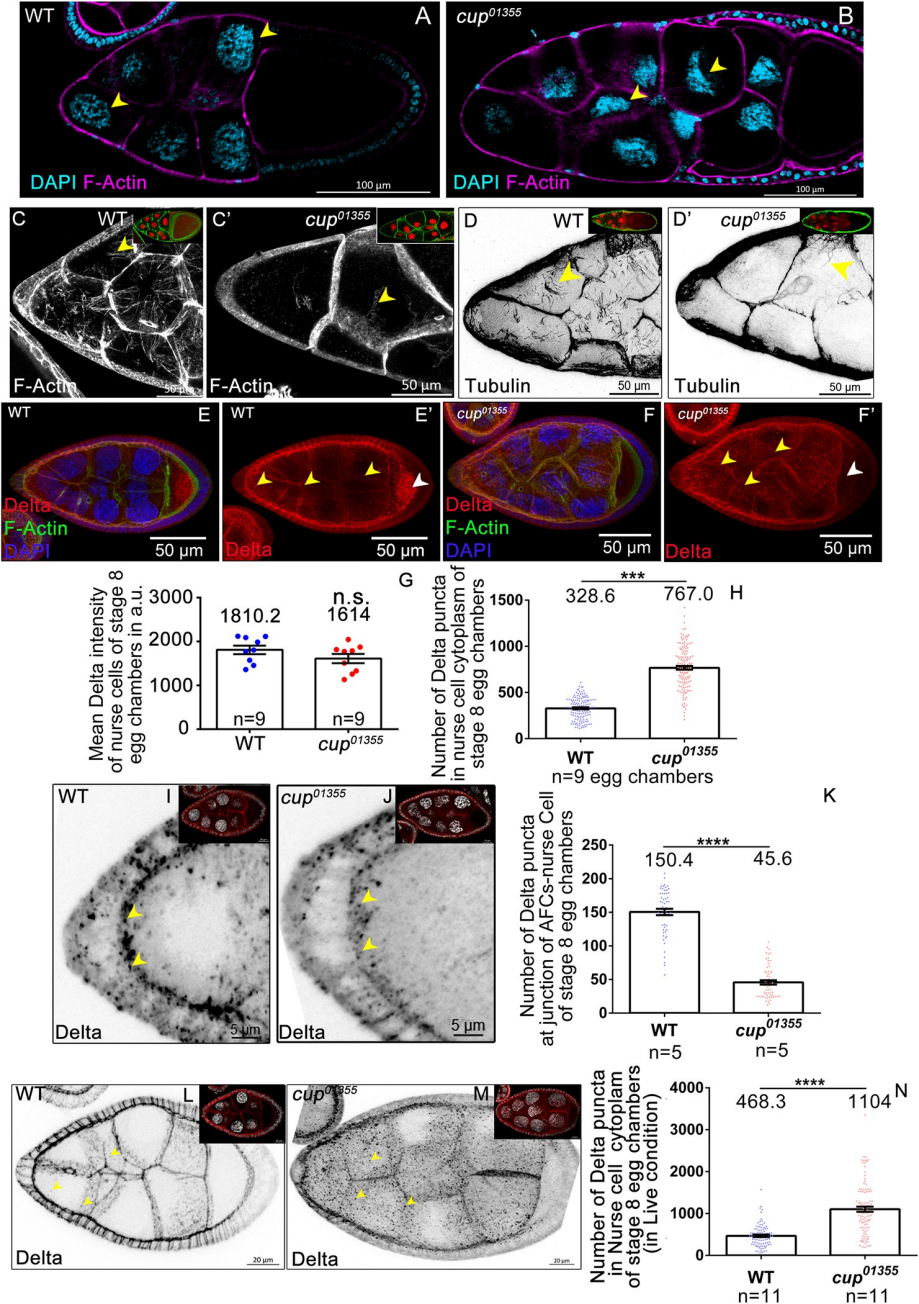
Cup modulates nurse cell cytoskeleton and Delta trafficking. **(A-B)** Nurse cell nuclear morphology is disrupted in *cup*^*01355*^ egg chambers. Nuclei (yellow arrow heads) are elongated and mispositioned in *cup*^*01355*^ egg chambers compared to round nuclei observed in wild type, F-actin (Magenta), DAPI (cyan). **(C-C’)** Phalloidin staining of *cup*^*01355*^ egg chambers exhibit reduced actin fibers (yellow arrow heads) compared to wild type egg, F-actin (grey, green in inset), DAPI (red). **(D-D’)** Tubulin stained *cup*^*01355*^ egg chambers show smaller, randomly distributed tubulin fibres (yellow arrow heads) in nurse cell cytoplasm compared to distinct radially arranged fibers observed in the wild type, tubulin (black, green in inset), DAPI (red). **(E-H)** Delta stained *cup*^*01355*^ egg chambers exhibit more cytoplasmic puncta in nurse cells as compared to wild type (yellow arrows). Oocyte Delta localisation is absent in *cup*^*01355*^ egg chambers as observed in the wild type (white arrow head). Delta (red), F-actin (green), DAPI (blue). Mean Delta intensity of wild type and *cup*^*01355*^ egg chambers is similar **(G).** However, the number of cytoplasmic puncta in the *cup*^*01355*^ nurse cells is higher than that observed in the wild type **(H)**. **(I-J)** Stage 8 egg chamber of indicated genotypes stained for Delta (black) (yellow arrowhead). Inset is the representative egg chamber. **(K)** Quantification of Delta puncta at the junction of nurse cell and anterior follicle cell suggest that Delta trafficking is perturbed in the cup mutants. **(L-N)** Delta endocytosis assay has shown that *cup*^*01355*^ egg chambers exhibit more cytoplasmic puncta (yellow arrow heads) in the nurse cells as compared to wild type (yellow arrow heads), Delta (black, red in inset), DAPI (white). n represents the number of egg chambers evaluated. SEM represent the error bars.

Decrease in the levels of NICD and NECD is indicative of inefficient Notch proteolysis which results in compromised signaling [50,51]. To analyze underpinnings of these alterations, we wondered if these changes can be correlated with the nurse cell morphology. Consistently, unlike the normal round shaped nurse cell nuclei in the WT, we observed elongated, mispositioned nurse cell nuclei in *cup*^*01355*^ egg chambers (Fig 4A and 4B). Since mispositioned nurse cell nuclei have been reported when the cytoskeleton is disorganized, we also examined the status of the actin cytoskeleton in *cup*^*01355*^ mutant egg chambers [52]. We stained the egg chambers with rhodamine-phalloidin and observed reduced levels of F-actin fibers in the nurse cells of *cup*^*01355*^ egg chambers unlike the control (Fig 4C and 4C’). In addition, we observed very sparse Tubulin fibers in the germline cells of *cup* mutant egg chambers compared to the control (Fig 4D–4D’). Together these changes suggested that aberrant nurse cell cytoskeleton could be one of the reasons for the disorganized germline and mispositioned nuclei seen in the *cup* mutant egg chambers.

Delta ligand is known to activate Notch in the follicle cells of developing egg chambers. Since cytoskeleton is critical for Delta trafficking and Notch activation, we next focussed our attention on the distribution Delta ligand in the nurse cells of *cup* mutant egg chambers [53]. First, we compared the levels of total Delta protein in the nurse cells of WT and *cup*^*01355*^ egg chambers. We did not observe any significant difference in the mean Delta intensity in nurse cells between WT (1810.22±97.63 SEM, n = 9 egg chambers) and *cup*^*01355*^ homozygous egg chambers (1614.43±105.29 SEM, n = 9 egg chambers) (Fig 4E–4F’ and 4G). Interestingly, however, the asymmetric posterior localization of Delta protein in WT oocytes was absent in the *cup*^*01355*^ mutant egg chambers (Fig 4E–4F’). Strikingly, we observed a large number of Delta positive puncta in the cytoplasm of nurse cells of *cup*^*01355*^ mutant egg chambers (767± 20.51 SEM, n = 9 egg chambers) unlike the control (328.6±10.35 SEM, n = 9 egg chambers) (Fig 4F–4G’ and 4H). *cup*^*8*^*/ cup*^*01355*^egg chambers also showed a similar increase (WT-341.4 ±30.3 SEM n = 5, *cup*^*8*^*/cup*^*01355*^- 747.5±47.2 SEM, n = 4) (S5A–S5C Fig). In addition, we observed very few Delta positive puncta at the apical interface of AFC and germline nurse cells (control—150.4± 4.68 SEM, n = 5; *cup*^*01355*^- 45.69±3.2225 SEM, n = 5) (Fig 4I–4J and 4K). Incidentally, it is the anterior-most FCs that acquire the migratory BC fate as oogenesis progresses. Delta being a transmembrane protein, its enrichment in the cytoplasmic fraction of the *cup* mutant nurse cells and its absence from the apical interface of AFCs suggested that Delta trafficking is probably perturbed in the *cup* mutants. As proper trafficking of Delta ligand in the nurse cells is critical for Notch activation, we decided to analyze it further [34].

### Delta endocytosis is impaired in cup mutants

Delta internalization by endocytosis in the signal sending i.e. ligand-producing cells is important for the activation of Notch signaling in the receiving i.e. receptor-expressing cells [53–55]. As the accumulation of Delta puncta in the nurse cells can be an outcome of either defective endocytosis or exocytosis, we first examined the status of these two processes in the *cup* mutant nurse cells. To this end, we carried out an Delta endocytosis assay on egg chambers with an antibody that recognizes the extracellular domain of Delta ligand (c594.9B). In live samples, the c594.9B antibody can bind only the extracellular fraction of total Delta ligand while the intracellular Delta fraction remains unbound. During the chase, Delta is internalized and moves through the endocytotic vesicles, so does the labelled fragment. Thus, in the endocytosis assay, the enriched Delta puncta observed in the *cup*^*01355*^ mutant nurse cells will be labelled if there are defects in endocytosis. While any aberrations in exocytosis will not result in accumulation of labelled fragment in the nurse cell cytoplasm [56,57]. When we conducted this experiment, we observed a conspicuous apical enrichment and a few randomly distributed cytoplasmic puncta of Delta in the follicle cells of both the WT and *cup* mutant egg chambers. Strikingly however, unlike the WT, we observed a significantly higher number of cytoplasmic Delta in the nurse cells of *cup*^*01355*^ mutant egg chambers similar to what was observed in fixed sample analysis (Delta particle count: WT- 468.3±27.84 SEM n = 11, *cup*^*01355*^*-* 1104±60.88 SEM n = 11) (Fig 4L–4N). As the cytoplasmic Delta in the *cup*^*01355*^ mutant nurse cells was significantly labelled in the endocytosis assay, it suggested that defects in Delta trafficking observed in the *cup*^*01355*^ mutants were predominantly due to impaired endocytosis. Given that the endocytic pathway of Delta trafficking is perturbed, we sought to identify which specific component of endocytosis may be responsible for this behaviour.

### Elevating Rab11 activity limits border cell fate

**Fig 5:**
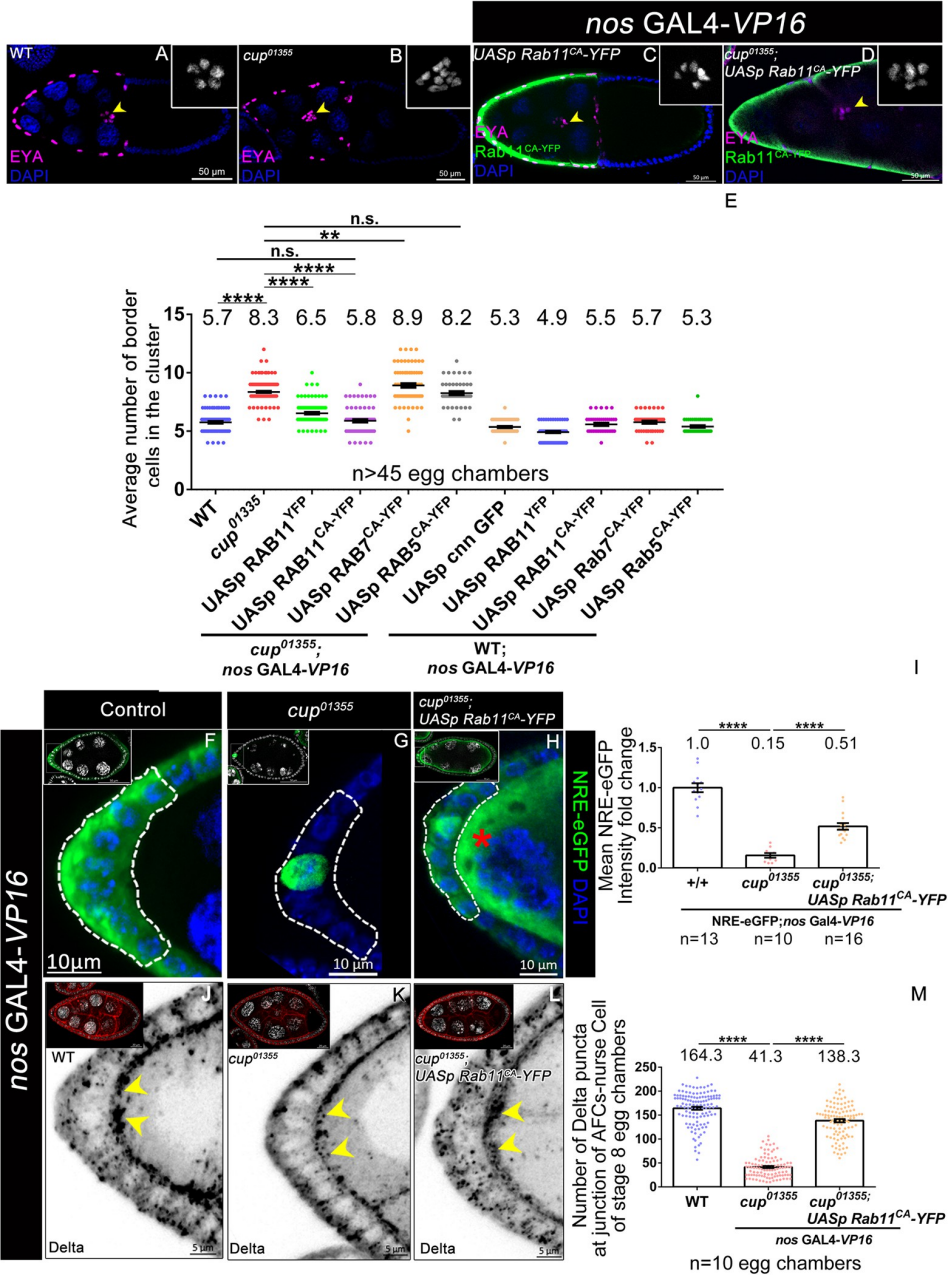
Overexpression of Rab11CA limits the border cell fate in the Cup mutant. **(A-E)** Stage 10 egg chambers of indicated genotypes stained with EYA (magenta), DAPI (blue, grey in inset) and YFP (green). yellow arrowheads mark the border cell cluster. Number of border cells is rescued when *Rab11*^*CA*^ is overexpressed in the nurse cells of *cup*^*01355*^ egg chambers. **** indicates a p value <0.0001 (Student t-test). ** indicates a p value <0.01 (Student t-test). “ns” indicates statistically not significant. **(F-I)** Stage 8 egg chambers of indicated genotypes. White dotted line outlines the Anterior follicle cells. NRE-eGFP (green) and DAPI (blue, white in inset). **(I)** NRE-eGFP intensity fold change with respect to the control is partially rescued when *Rab11*^*CA*^ is overexpressed in nurse cells of *cup*^*01355*^ egg chambers as compared to *cup*^*01355*^ mutant egg chambers alone. Red asterisk in nurse cells indicates tagged YFP expression when *Rab11*^*CA*^ is over expressed with germline specific *nos* GAL4*- VP16*. **** indicates a p value <0.0001 (Student t-test) **(J-M)** Stage 8 egg chambers of indicated genotypes. Yellow arrowheads mark the Delta puncta at the junction of anterior follicle cell and nurse cells, Delta (black, red in inset), and DAPI (white). **(M)** Quantification indicates that Number of Delta particles at the junction of anterior follicle cell and nurse cells is partially rescued when *Rab11*^*CA*^ is overexpressed in the nurse cells of *cup*^*01355*^ egg chambers as compared to that observed in *cup*^*01355*^ egg chambers alone. **** indicates a p value <0.0001 (Student t-test). n represents the number of egg chambers evaluated and SEM represent the error bars in each panel.

Endocytosis is a multi-step process wherein the fate of the internalized cargo is decided in the early endosomes between either recycling or degradation [58–61]. Rab5GTPase plays a crucial role in the biogenesis of endosomes and aids in the maturation of early endosomes to late endosomes. While Rab11GTPase facilitates the recycling of the cargo from the early endosomes to the plasma membrane [62–64]. The cargo marked for degradation moves from the late endosome to the lysosome with the help of the activity of Rab7GTPase [65]. We were curious if endocytosis of Delta ligand was impaired at a specific point during endocytosis in *cup* mutants. We thus wondered if overexpression of a specific constitutively active RabGTPase (CA) in the nurse cells of the *cup*^*01355*^ egg chamber could reduce the number of BCs in *cup* mutant egg chambers. We observed that overexpression of Rab11GTPase^CA^ in the *cup*^*01355*^ nurse cells rescued the number of BCs to the control levels (*cup*^*01355*^-8.36±0.081SEM, rescue-5.889±0.0.12 SEM, wild type-5.757±0.082SEM, n≥80 egg chambers) (Fig 5A–5E). However, we didn’t observe any significant difference in the BC numbers when Rab5GTPase^CA^ (8.260±0.1479) and Rab7GTPase^CA^ (8.913±0.1632) were over-expressed in the *cup*^*01355*^ nurse cells (Figs 5E and S6D–S6G). Neither did we observe any restriction of BC fate specification, when Rab11GTPase^CA^ was overexpressed in the WT germline nurse cells. In addition, unlike Rab11GTPase^CA^, we observed a modest rescue in the border cell fate in *cup* mutants overexpressing Rab11^WT^(*cup*^*01355*^-8.36±0.081SEM; *cup*^*01355*^; *UASp Rab11*^*YFP-WT*^-6.53±0.08 SEM n≥100 egg chambers) (Figs 5E and S6A–S6C). As specifically activating the recycling component of the endocytosis can restore the BC fate to near WT numbers in the *cup*^*01355*^ mutant egg chambers, it suggested that Rab11GTPase activity downstream of Cup modulates BC fate specification in the AFCs.

Next, we tested if the rescue is indeed due to the restoration of Notch signaling in the AFCs of *cup*^*01355*^ egg chambers upon overactivation of Rab11GTPase. We measured Notch reporter activity by quantifying NRE-eGFP levels, and observed a 0.5-fold upregulation of Notch activity when Rab11GTPase^CA^ was overexpressed in the nurse cells of *cup*^*01355*^ egg chambers as compared to that of control (*cup*^*01355*^ mutant egg chambers) (WT- 1.00±0.05 SEM, n = 13, *cup*^*01355*^- 0.16±0.028SEM, n = 10, rescue- 0.52±0.040 SEM, n = 16) (Fig 5F–5I). We also observed rescue in the number of Delta puncta at the apical interface of AFC and germline cells of Cup depleted egg chambers that were over-expressing Rab11^CA^-YFP(wild type- 164.3±3.125 SEM, n = 10, cup01355- 41.39±2.16SEM, n = 10, rescue- 138.3±3.396 SEM, n = 10) (Fig 5J–5M). Irrespective of partial rescue in the levels of NRE-eGFP and Delta puncta count, we noticed a complete reversion of border cell numbers when Rab11GTPase^CA^ was overexpressed in the *cup* mutant germline. This may suggest that BC fate specification is quite robust beyond a certain level of signaling or that Cup may affect other aspects of egg chamber development independent of Rab11 function.

The rescue of border cell numbers and Delta puncta in *cup* mutant upon over-expression of Rab11GTPase^CA^ was clearcut. We were thus curious whether such a rescue was possible despite the disruption of cytoskeleton. When we examined the status of actin and tubulin upon overexpression of Rab11GTPase^CA^ in nurse cells, we observed restoration of actin filaments in the *cup* mutant nurse cells like WT (S6H–S6K Fig). However, we didn’t see any rescue of aberrations in tubulin distribution. Altogether, these results suggest that Rab11GTPase^CA^ overexpression rescues the actin filament structure which may help Delta trafficking and appropriate activation of Notch in the AFCs. However, over-expression of either actin or tubulin or both in cup mutant nurse cells failed to restrict the increase in border cell count observed in the *cup* mutant egg chambers (S5D Fig). Altogether these data underscore the importance of Rab11 downstream of *cup* during BC specification and also argue that there may be other additional targets downstream of Rab11. Next, we were curious to examine the role of Rab11 in border cell fate specification with a specific focus on Delta trafficking.

### Germline Rab11 affects Delta trafficking and border cell fate in the anterior follicle cells

**Fig 6:**
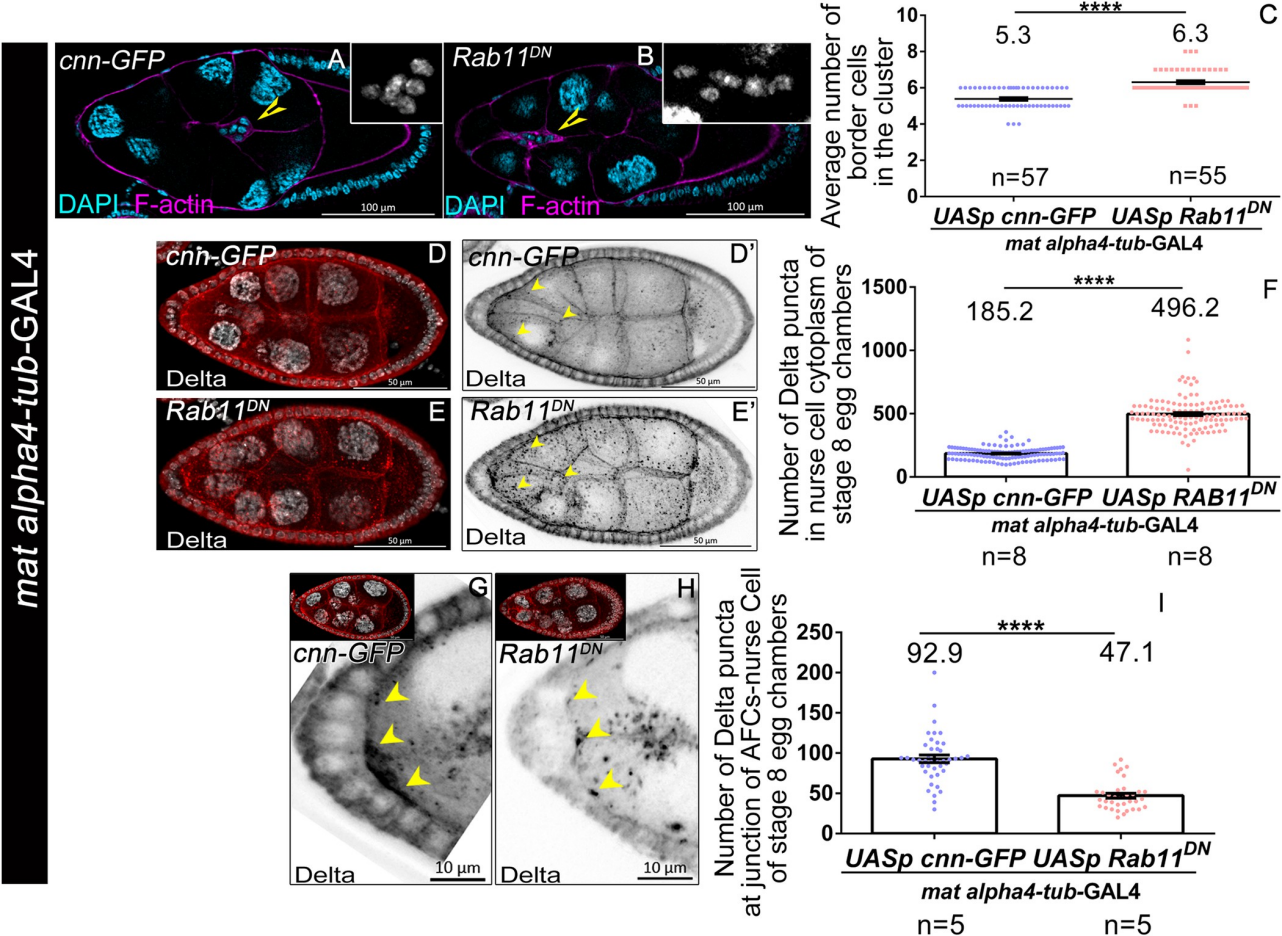
Rab11 function in the germline modulates Delta Trafficking and Border cell specification from the follicle cells. **(A-C)** Downregulation of Rab11 activity by over-expressing *Rab11*^*DN*^ in the nurse cells results in increased Border cell count compared to control (*mat alpha4-tubulin-*GAL4; *UASp-cnnGFP*). Yellow arrow heads mark border cell cluster, F-actin (magenta), DAPI (cyan, grey in inset). **(D-F)** Overexpression of *Rab11*^*DN*^ in nurse cells using *mat alpha4-tubulin-*GAL4 exhibits cytoplasmic Delta puncta (yellow arrows), Delta (red) in D and E, Delta (black) in D’ and E’. DAPI (blue), GFP (green). **(G-I)** Overexpression of *Rab11*^*DN*^ in the nurse cells with *mat alpha4-tubulin-*GAL4 exhibits reduced Delta puncta at the junction of anterior follicle cells and nurse cells, Delta (black, red in inset), DAPI (grey in inset). **** indicates a p value <0.0001 (Student t-test). n represents the number of egg chambers evaluated in each panel.

Given that increasing Rab11 activity rescues the border cell fate in *cup*^*01355*^ egg chambers, we were curious if manipulating Rab11 activity can influence Delta trafficking and border cell fate specification on its own. To test this, we downregulated Rab11 function in nurse cells by expressing a dominant negative construct of Rab11 that inhibits GTP binding [66]. We employed *mat alpha4-tub-*GAL4 to bypass the early requirement of Rab11 and targeted its downregulation around mid-oogenesis. Satisfyingly, we observed that functional depletion of Rab11 in the nurse cells resulted in a higher number of AFCs acquiring BC fate as compared to the controls (*Rab11*^*DN*^- 6.3± 0.08 SEM, n = 55; *cnn-GFP*- 5.3± 0.07 SEM, n = 57) (Fig 6A–6C). The modest increase in the number of cells that acquire BC fate could be attributed to the corresponding level of overexpression of *Rab11*^*DN*^ construct. Nevertheless our results suggest that Rab11 activity in the nurse cells modulates BC fate in the AFCs in a non-cell autonomous manner. Next, we examined the status of Delta in Rab11 depleted nurse cells and observed that the number of cytoplasmic Delta puncta was much higher than the control (*Rab11*^*DN*^- 496.5± 12.05 SEM, n = 8; *control*- 185.2± 4.51 SEM, n = 8) (Fig 6D–6F) In addition, we observed less number of Delta puncta at the apical interface of AFC and germline nurse cells similar to what we observed in *cup* mutants (*Rab11*^*DN*^- 47.1± 3.1 SEM, n = 5; *cnn-GFP*- 92.91± 4.6 SEM, n = 5) (Fig 6G–6I). Altogether these results reinforce our claim that Rab11 activity downstream of Cup non-cell autonomously limits the border cell fate in the AFCs.

Next, we were curious to examine how Cup may regulate the Rab11 activity in the nurse cells to influence the BC fate specification from the AFCs.

### Cup mutants exhibit Rab11 enrichment in the trans-Golgi compartment

Given that Rab11 seems to be downstream of Cup in modulating BC fate, we wondered how Cup modulates Rab11 function. To examine this, we employed the protein trap line Rab11 (EYFP) wherein YFP^MYC^ is tagged to the endogenous Rab11 [67]. We observed intense staining in follicle cells and a relatively diffuse staining in the nurse cell cytoplasm of both WT and Cup mutant egg chambers. Thus, Rab11 levels are not significantly affected in the *cup*^*01355*^ mutant background.

**Fig 7:**
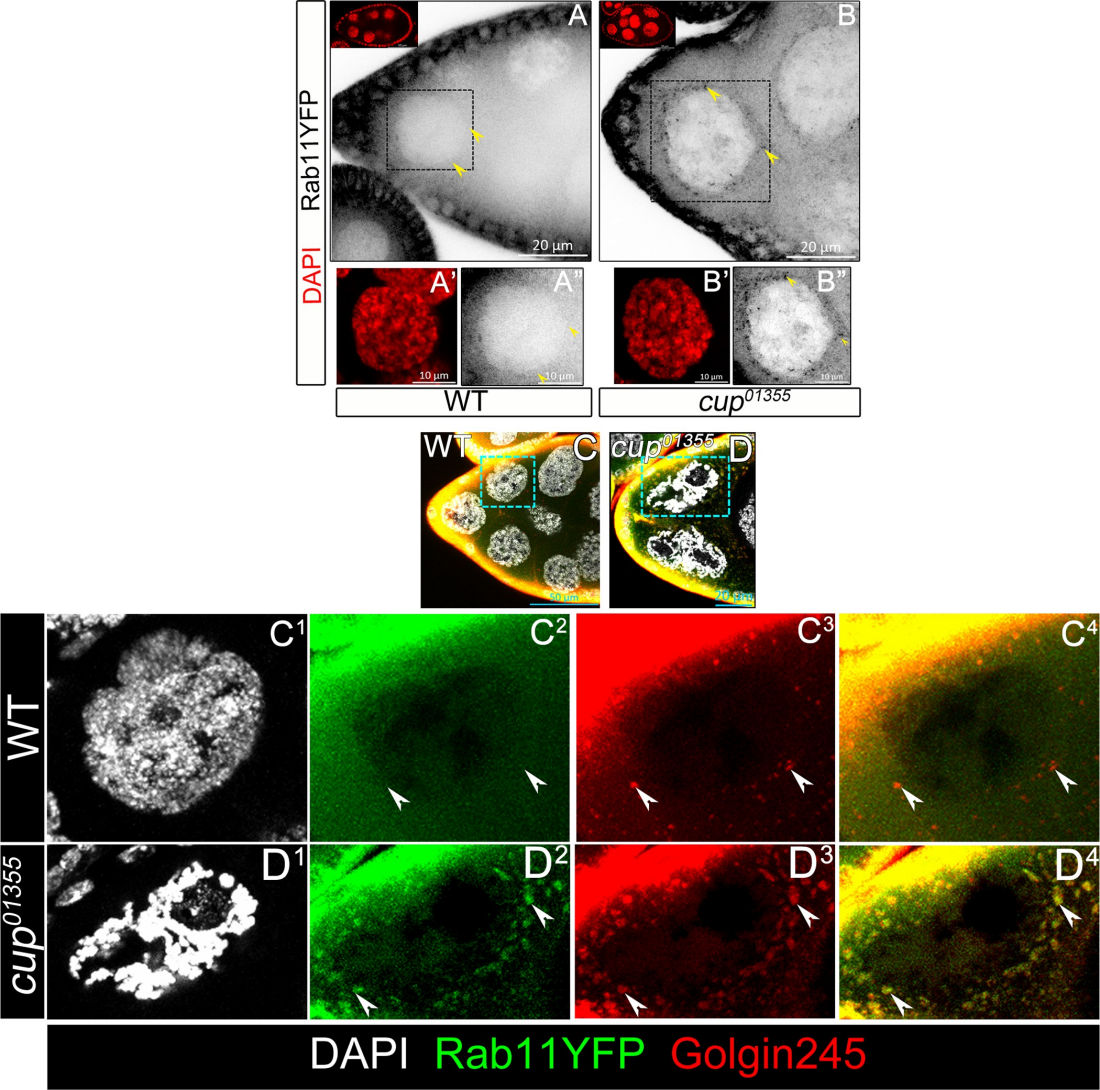
Cup mutants exhibit Rab11 enrichment in the trans-Golgi network. **(A-B”)** Rab11-YFP puncta is enriched in the nurse cell peri-nuclear zone of *cup*^*01355*^ egg chambers. A’,A” and B’, B” are the magnified square region outlined in A and B respectively. Rab11-YFP (black) indicated by yellow arrowhead, DAPI (red). **(C-D, C**^**1**^**-D**^**4**^**)** Rab11-YFP colocalizes with trans-Golgi network marker Golgin-245 at periphery of nurse cell nucleus in the *cup*^*01355*^ egg chambers. Rab11-YFP (green) indicated by white arrow heads, Golgin-245 (red) and DAPI (white).

Strikingly we observed a punctate distribution of Rab11 around the perinuclear region of the nurse cells in the *cup*^*01355*^ mutant egg chambers that was absent in the control samples (Fig 7A–7B”). This observation prompted us to check the intracellular localization of Rab11 puncta. Rab11 has been shown to exhibit differential localization based on its activity within a cell and golgi serves as the site where nucleotide exchange facilitates Rab11 activation [68]. We stained the Rab11 YFP expressing egg chambers with Golgin 245 antibody that is known to mark the trans-Golgi network [69]. We observed several Golgin-245 puncta similar to that we observed for Rab11YFP in the *cup* mutant egg chambers. Strikingly, in the *cup*^*01355*^ mutant germline cells, substantial number of Golgin-245 puncta colocalized with the Rab11YFP suggesting that Rab11 is enriched in the trans-Golgi network (Fig 7C–7D and 7C^1^–7D^4^).

Altogether our data suggest that Rab11 activity is diminished in the *cup*^*01355*^ egg chambers. This may be due to the inability of the Rab11 to undergo nucleotide exchange in the *cup*^*01355*^ mutants thus trapping them in the trans-Golgi network. Consistently, stimulating the recycling endocytosis in the nurse cells of the *cup*^*01355*^ egg chambers restores Notch signaling in the AFCs thus limiting JAK-STAT activation and restricting BC cell fate specification.

Altogether, our results suggest that interaction between germline nurse cells and overlying anterior follicle cells regulates the migratory fate of the border cells. Specifically, our data suggest that recycling of Delta ligand in germline nurse cells aids in Notch activation in the anterior follicle cells, thus restricting the domain of JAK-STAT signaling in the AFCs. In Cup mutants, Delta recycling is impeded, thus compromising Notch activation and resulting in excessive STAT, transforming a higher number of AFCs to migratory BC fate. Altogether, it appears that Cup protein modulates Delta recycling by regulating Rab11 activity in the germline nurse cells, which subsequently aids in non-cell autonomous Notch activation in the AFCs. Once Notch is activated, it restricts JAK-STAT signaling in the FCs, thus optimizing the number of cells acquiring BC fate. Our data thus provides a novel insight into how the communication between germline and soma may regulate cell fate specification during development.

## Discussion

**Fig 8:**
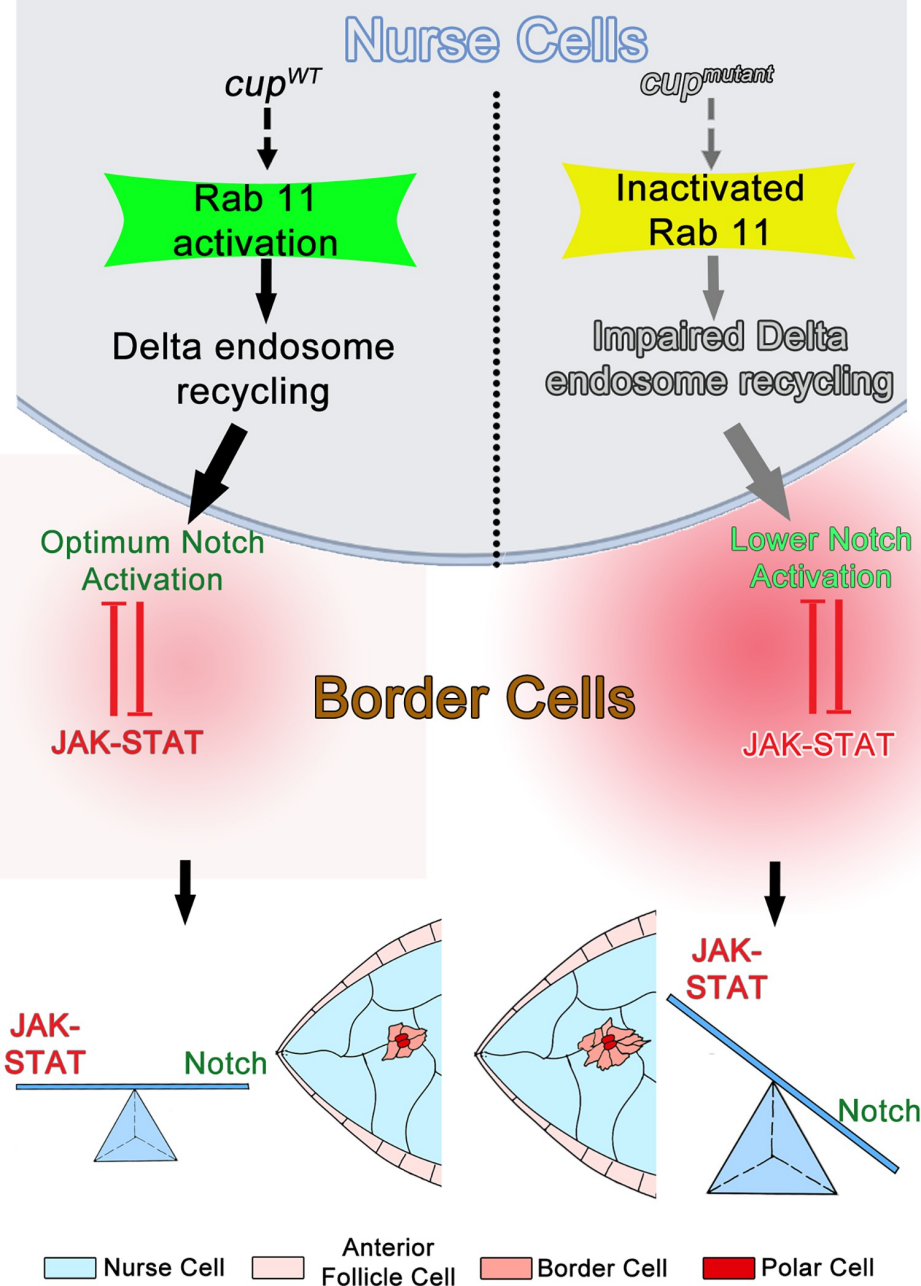
Schematic of Proposed Model. Cup function potentiates Delta recycling in the germline nurse cells. This stimulates non-cell autonomous activation of Notch signaling in the Anterior Follicle cells (AFCs). As Notch and JAK-STAT are antagonistic, a balance between these two signaling cascades aids in transformation of an optimum number of stationary AFCs to migratory border cell fate. In the *cup* mutants, Notch signaling is impeded, which results in higher levels of STAT and larger number of AFCs acquiring migratory border cell fate.

Cell fate specification is the fundamental basis of cellular diversity in developing metazoans. Among the diverse mechanisms underlying generation of distinct cell fates, intercellular communication occupies a central position. Delineation of the migratory individuals from a stationary population is one of the important outcomes of such cellular communication. Directed cell migration plays an important role both in normal development and as well as in various disease conditions including tumor metastasis. Since cell migration is critical for both normal and pathological events, we have employed the *Drosophila* oogenesis model to analyze how intercellular communication between germline and soma results in the specification of BCs from the AFCs. So far, we know that BC fate acquisition is under the strict surveillance of signalling between the somatic FCs. Here we report a novel control mechanism involving germline-soma interaction that limits the size of the migratory BC cluster. We show that the RNA binding protein, Cup, maintains the nurse cell cytoskeleton and regulates Delta trafficking in the germline cells thus potentiating Notch activation in AFCs. In the absence of Cup function, Notch signalling is mitigated, leading to elevated STAT and as a result an excess number of AFCs acquire the BC fate (Fig 8)

This study was carried out using a hypomorphic allele for Cup, which exhibits morphological defects during mid-oogenesis. Given that the Cup gene product plays an essential role in early oogenesis, using this partial loss of function allele helped us bypass the earlier requirement of Cup and thus, allowed the examination of the role of Cup during vitellogenesis. In general, our study highlights the importance of hypomorphic alleles for studying the temporal requirement of gene products that exhibit pleiotropic effects during development.

Importantly, our data underscores how careful regulation of Notch-Delta signaling helps acquisition of migratory fate and how the local environment can control the total number of cells that acquire this trait. Though interaction between the germline and follicle cells has been reported to affect epithelial morphogenesis, this is the first report where germline derived Cup protein is shown to non-cell autonomously limit BC fate by restricting Notch signaling in the AFCs. The expression patterns of Notch reporter *m7*-Lacz and STAT overlap in early stage 8 egg chambers and our data suggest that Notch and JAK-STAT are functionally antagonistic in mid oogenesis as it was reported during early stage egg chambers [33]. We speculate that this interaction assists in the formation of a migrating BC cluster of the adequate size. Subsequently, the non-motile polar cells that assists in the formation of a functional micropyle, reach the oocyte boundary in conjunction with the BCs.

Our data raises an obvious question about mechanism of action of Cup protein that limits border cell fate in the developing egg chamber. Cup may regulate germline cytoskeleton to influence Delta ligand trafficking and thus indirectly affecting BC fate specification. However, overexpression of Actin and/or Tubulin alone or together failed to rescue the *cup* mutant phenotype. Thus we favor the possibility that Cup affects the cytoskeletal dynamics in developing egg chambers. In conjunction with our results with the available data on activities of Cup, it likely performs a diverse set of functions in the nurse cells ranging from affecting stability of the cytoskeleton to modulate Delta trafficking to regulating the output from maternal mRNAs. Future studies will focus on how diverse functions of Cup are regulated and how its activity is differentially calibrated to achieve proper development of germline.

The other unresolved question concerns how Notch and STAT signaling intersect during these processes. Previous reports indicate that expression patterns of Notch reporter, *E(spl)m7*-Lacz, and STAT overlap both in the AFCs from the early stage 8 egg chambers and in migrating BCs [33]. Nonetheless, our data suggest that Notch and STAT function antagonistically downstream of Cup to mediate border cell fate from the AFCs. A similar relationship between Notch and STAT has been reported in the early stages of oogenesis [33]. In this regard it is interesting to note that Broad mutants exhibit higher levels of STAT in the follicle cells of stage 10 egg chambers [70,71]. Interestingly, Broad is a known transcriptional target of Notch in the follicle cells, which is consistent with the possible antagonistic activities of Notch and STAT in the follicle cells [71]. Taken together with the overlap in expression patterns of *E(spl)m7*-Lacz and STAT we hypothesize that JAK-STAT and Notch mutually fine tune activities to ensure that optimum number of BCs are specified from the AFCs. Consequently it will be interesting to elucidate the mechanism of how Notch and JAK-STAT signaling pathways regulate their respective activities

Furthermore, Notch signalling is essential during mid- oogenesis and our data suggest that recycling of Delta is critical for this aspect of Notch function. Thus far, two different models have been proposed to link Delta trafficking with Notch activation. The first model posits that the pulling force generated by Delta-Notch endocytosis in the ligand-producing cell facilitates S2 cleavage of the Notch receptor, thus activating Notch signaling in the signal-receiving cells. While the alternate model proposes that Delta endocytosis, coupled with recycling, facilitates interaction between Delta and the Notch receptor thus activating Notch signaling in the receptor-producing cells [45]. In our study, activating the recycling component of endocytosis in the nurse cells restored Notch signaling and mitigated the excess specification of BCs in the Cup mutant egg chambers. Thus, our data suggest that Delta recycling in the signal producing cells is critical for Notch activation in the adjacent AFCs during mid-oogenesis. In future, it would be worth examining how Cup modulates Delta recycling in the germline cells of developing egg chambers.

Notch signaling is a evolutionarily conserved pathway and is known to regulate several aspects of metazoan development. Thus, it would be worthwhile to examine if germline–soma communication mediated by Notch signaling operates in higher systems [72,73]. Of note, misregulation of Notch and STAT signaling is associated with several solid tumors. It would be interesting to investigate if imbalance between the two signaling networks may underlie tumorigenesis, metastasis and tumor progression.

## Materials and methods

### *Drosophila* stocks and crosses

Fly stocks and crosses were maintained at 25°C and were incubated at 29°C during GAL4 based experiments. The *cup* alleles, *cup*^*01355*^ (BL-12218), and *cup*^*15*^ (BL-29718) were obtained from the Bloomington Stock Centre (BDSC) These two alleles have been characterized by Keyes and Spradling, and *cup*^*8*^ was gifted by Prof. Trudi Schüpbach. Western blot analysis of *cup*^*01355*^ ovaries shows >50% reduction in Cup protein level as compared to wild type [38]. The *cup*^*15*^ allele is stronger and has been generated by EMS mutagenesis. Western blot analysis of *cup*^*15*^ ovaries shows a very negligible amount of Cup protein as compared to the wild type [38].

For the over expression Cup in the germline, a pUASp-Cup expression transgenic fly line was generated at the Centre for Cellular And Molecular Platforms (C-CAMP) facility, in Bangalore, India. Cup-CDS construct from the BDGP clone LD47924 (Berkeley Drosophila Genome Project), was cloned in the pUASp vector and the construct was used for microinjection. *nos*. *NGT* GAL4 {Bloomington Stock Center (BDSC 25751)} and *nos* GAL4-*VP16* {Bloomington Stock Center (BDSC 7253)} were used for expressing various transgenes in the germline. The *upd*-lacZ fly stock was a kind gift from Prof. Henry Sun. The stock is generated by inserting a P{lacW} 2851 bp upstream of the 5’ end of upd1 [74]. This construct acts as an enhancer trap reporter enzyme which also harbours a nuclear localization signal. This expression of lacZ reflects the transcription based on the enhancer activity of the endogenous *upd1* gene. This construct does not reflect the translation status of Upd.

*cup* RNAi (BL-35406),) Notch response reporter line (BL-30727), UASp GFP-Cnn (BL-7255), UASp capu.GFP (BL-24763) UASp Rab11-YFP (BL-9790), UASp Rab11^CA^-YFP (BL-9791), UASp Rab5^CA^-YFP (BL-9773), UASp Rab7^CA^-YFP (BL-50785), slbo Gal4 (BL 58435), Rab11 PTT (BL 62549), were obtained from BDSC. UAS NICD, *mat alpha4-tub-*GAL4*-VP16* (*mat alpha4-tub-*GAL4) was gifted by Daniel St. Johnston. For MARCM experiments the stock P{ry[+t7.2] = hsFLP}1, y[1] w[*] P{w[+mC] = UAS-mCD8::GFP.L}Ptp4E[LL4]; P{w[+mC] = tubP-GAL80}LL10 P{ry[+t7.2] = neoFRT}40A; P{w[+mC] = tubP-GAL4}LL7 (BL-42725) was used to cross with *cup*^*15*^ FRT 40A fly stock. BL-5192 was used to recombine FRT40A with *the cup*^*15*^ allele. Canton-S flies were used as wild-type control flies.

For MARCM experiments 1–2 days old F1 flies were collected and incubated at 37°C for 1 hour, three times a day with a minimum two-hour interval in between the subsequent heat shocks. Heat shock was given for three consecutive days and flies were fattened at 25°C after 5 days for 20–22 hrs and then dissected. *cup*^*15*^ homozygous mutant follicle cell clones which spanned the entire anterior end of the egg chamber including the whole border cell cluster were used for quantification of the number of border cells in the cluster.

For GAL4 expression-based experiments, 2–3 days old flies were incubated at 29°C for 20 hours followed by dissection. For mutant-based experiments, 2–3 days old flies were incubated at 25°C for 20 hours followed by dissection.

For downregulation of Cup in the germ line we crossed *mat alpha4-tub*-GAL4 with Cup^*RNAi*^. The crosses were set up at 25°C. In the early pupal stage, the vials were transferred to 20°C. 2days old eclosed flies were fattened at 20°C for 15 hours followed by 5 hours at 25°C and then dissected.

### Immunostaining

Ovaries were dissected in Schneider’s media containing 10% FBS (Foetal Bovine Serum, US origin, catalog no. 16000044) and fixed with 4% p-Formaldehyde (Sigma-Aldrich, catalog no. 158127) for 15 minutes at room temperature. Blocking was done with 1X PBS (Sigma-Aldrich, catalog no. P3813) containing 0.3% Triton X-100 (Affymetrix, catalog no. T1001) and 5% BSA (Bovine Serum Albumin, Amresco, catalog no. 0332) for 1hour at room temperature. Mouse anti-Cup antibody was gifted by Prof. Akira Nakamura and used at 1: 10000 dilutions. Rat anti- Slbo antibody was gifted by Prof. Pernille Rorth and used at 1:500 dilutions. Rabbit anti-STAT was gifted by Steven Hou and used at 1:750 dilutions. Mouse anti-α-Tubulin antibody (T9026) was obtained from Sigma and used at 1:600 dilutions. Mouse anti-Armadillo (N27A1), mouse anti-Fas III (7G10), mouse anti-Delta (C594.9B), mouse anti-FasII (1D4), mouse anti-EYA (10H6) mouse β-Gal (40-1a) and goat-golgin245 were obtained from Developmental Studies Hybridoma Bank (DSHB) and used at 1:100, 1:500, 1:200, 1:100, and 1:200 dilutions respectively. Phosho-histone 3 antibody (Cell Signaling Technology, 9713S) was used at 1:150 dilutions. Rabbit anti-GFP (A-11122, Invitrogen) was used at 1:1500 dilutions. Secondary antibodies conjugated with anti-mouse, anti-rabbit, and anti-goat Alexa-488 and Alexa-568 (Molecular Probes) were used at 1:400 dilutions.

For Tubulin cocktail(α+β) staining, Individual egg chambers were dissected in 1X PEM buffer (60mM PIPES, 25mM HEPES, 10mM EGTA, 4mM MgSO_4,_ pH = 6.8) and fixed with 10% formaldehyde in presence of 1X BRB80 buffer [75] and 1% Tween 20 (Amresco). After fixation, the samples were incubated with 1X PBS containing 1% Triton X-100 and 1X BRB80 buffer overnight at 4°C. Blocking was done with 1X PBS (Sigma-Aldrich, catalog no. P3813) containing 1% Triton X-100 (Affymetrix, catalog no. T1001) and 5% BSA (Bovine Serum Albumin, Amresco, catalog no. 0332) and 1X BRB80 buffer for 4 hours at room temperature. Mouse anti-alpha-tubulin (T9026) was obtained from Sigma and used at 1:800 dilution and anti-beta-tubulin (E7) was used at 1:200 dilution. Incubation of the primary antibody cocktail was carried out overnight at 4°C. The sample was washed with 1X PBS containing 0.5% Tween 20, Followed by incubation in a secondary antibody conjugated with Alexa-488 mouse or Alexa-568 mouse used at 1:400 dilutions.

### Measurement of the size of the border cell cluster

For measuring the volume, completely detached border cell clusters at stage 9 that have not reached the oocyte boundary have been considered for analysis.

The border cell clusters are generally spheroid in structure. To measure the size of the cluster, the whole cluster was imaged at 40X magnification taking z stacks at optimal Z intervals suggested by the Zen 2012 software. The image processing and analysis were done using Zen 2012 software. All the stacks were merged to obtain a 2-dimensional maximum intensity projection (MIP) image. The cluster was outlined in the MIP image, the maximum and minimum diameter of the cluster was drawn, and the length obtained from the software was noted. The minor and major axes of the spheroid cluster were obtained by dividing the maximum and minimum diameters by 2 respectively. The Border cell cluster volume was obtained using the formula for spheroid (4/3π a^2^b, where a is the major axis, and b is the minor axis. Images were acquired in Zeiss Axio observer 7 with Apotome.2 module.

### STAT intensity quantification

To measure the STAT intensity, the anterior end of stage 8 egg chambers of wild type and *cup*^*01355*^ egg chambers were imaged with a center z section passing through the middle of the anterior polar cells. Z sections were captured at regular intervals for both kinds of samples. The exposure time was kept identical for image acquisition in DAPI (7.2 ms) and Rhodamine channel (4s) (for STAT signal acquisition) for control and experiment samples. All the stacks were merged to obtain a 2-dimensional maximum intensity projection (MIP) image, three nuclei on either side of anterior polar cells were outlined, and the mean STAT intensity was noted. Mean STAT intensity was calculated for each egg chamber. The average Mean intensity for the control and experimental samples was determined and subsequently plotted with statistical tests.

Images were acquired in Zeiss Axio observer 7 with Apotome 2 module and analyzed with Zen 2012 software.

### Notch response element GFP intensity calculation

For measuring the NRE-eGFP levels, the anterior end of stage 8 egg chambers was imaged keeping identical exposure time (GFP channel-400 ms) and other imaging parameters. Stage 8 was identified by depleted levels of Fas2 protein for both the control and the mutants [76]. A single follicle cell layer above and below the polar cell-containing layer was imaged taking z-sections at regular intervals. The z-planes were merged to obtain a 2D image, and four cells on either side of the polar cell along with the polar cells were outlined as the single region of interest in the anterior end of the egg chamber. The mean eGFP intensity of the main body follicle cells (4 cells) was used for background correction. The mean of the corrected eGFP intensity for the control and experimental egg chambers was plotted as fold change where we kept control as 1 with statistical tests. Images were acquired in Zeiss Axio observer 7 with Apotome.2 module and analyzed with Zen 2012 software.

### Quantification of nuclei in the border cell cluster

The nuclei were labeled using either an anti-Slbo antibody or DAPI. The stage 9 or 10 egg chambers, which had detached completely from the anterior end were considered for quantification. The number of border cell nuclei except the two polar cells was counted for every cluster, and the value was plotted. For counting Slbo-positive cells, all the nuclei in the cluster, including the polar cells, were counted. In all other experiments, wherever DAPI or EYA was used to evaluate the number of border cell nuclei, the polar cell nuclei were excluded based on their smaller size.

### Quantification of follicle cell nuclei of stage 8 egg chambers

The nuclei were labeled with DAPI. The oocyte size was used as a reference to select the stage 8 egg chamber. Images of whole egg chambers were acquired at regular z intervals of 0.43μm in a Zeiss LSM 710 confocal microscope and analyzed with Zen 2012 software. For each z plane, an ROI was selected and only nuclei that were in focus were counted. Overlapping nuclei across the Z planes were counted only once.

### RNA isolation and RT-PCR

The RNA isolation was done from the ovaries of adult flies using Trizol Reagent followed by cDNA preparation. The status of the Cup transcript was evaluated by RT-PCR.

Rp49:Forward-5’CTAAGCTGTCGCACAAATGGC3’,

Reverse- 5’AA CTTCTTGAATCCGGTGGGC3’,

Cup:Forward-5’AATCGTTGGGCCACATCCGA3’,

Reverse-5’TCATAGCCAACCGC CTGTGACT3’

Rp49 was used as a housekeeping control.

### Delta puncta quantification

To visualize the Delta distribution, the entire wild type and *cup*^*01355*^ stage 8 egg chambers were imaged taking z sections at regular intervals of 40X magnification in Zeiss LSM 710 confocal microscope or Zeiss Axio observer 7 with Apotome.2 module. For quantifying the nurse cell cytoplasmic Delta puncta, the z-sections encompassing the nurse cells were extracted. The z-section images at regular intervals of 0.68 μm were used for counting the puncta to avoid overlapping of puncta amongst the z planes. The particles were counted using the Image J software. The nurse cell area, excluding the follicle cells and oocyte, was outlined for every image. A threshold value was selected on the basis that each Delta puncta were detected as an individual spot, and the background was excluded. The particles were automatically counted by the <Analyze Particles> option. The average radius of Delta particles was measured and found to be within 0.5 μm. The particle size value range was set from 0.2–1.2 μm^2,^ and circularity was set from 0.5–1. The particles were counted for 9 egg chambers each of control and *cup*^*01355*^ and the total count of Delta particles for each plane was plotted with statistical tests.

For quantifying the Delta puncta at the anterior nurse cell-follicle junction, z sections encompassing the apical nurse cells-follicle cells junction were extracted. The apical membrane marked by Delta proteins was used as a reference to delineate follicle cells from the nurse cells. The z images at regular intervals of 0.68 μm were used for counting the Delta puncta. The particles were counted using the Image J software. The apical nurse cells-follicle cells junction was outlined for every image. A threshold value was selected on the basis that each Delta puncta were detected as an individual spot, and the background was excluded. The particles were automatically counted by the <Analyze Particles> option. The average radius of Delta particles was measured and found to be within 0.5 μm. The particle size value range was set from 0.2–1.2 μm^2,^ and circularity was set from 0.5–1. The particles were counted for 9 egg chambers each of control and *cup*^*01355*^ and the total count of Delta particles for each plane was plotted with statistical tests.

### Live Delta endocytosis assay

Individual egg chambers were dissected in live imaging media (LIM) [77]. After dissection, the egg chambers were incubated in LIM supplemented with mouse anti-Delta (1:20 dilution) for 1 hour at 25°C. After primary antibody incubation, the egg chambers were washed with LIM two times and fixed in 4% PFA for 15 mins. This was followed by the standard Secondary antibody staining. Secondary antibodies were conjugated with Alexa-488 or Alexa-568 (Molecular Probes) and were used at 1:400 dilutions.

### Delta intensity quantification

To quantify the total Delta protein, z-section images of the entire stage 8 control and *cup*^*01355*^ egg chambers (follicle cells and nurse cells) were acquired at regular intervals of 0.43μm in a Zeiss LSM 710 confocal microscope. The z planes were merged to obtain a 2D MIP image, and the whole egg chamber was outlined to determine the mean Delta intensity and plotted with statistical tests. Zen 2012 (blue edition) was used to analyze the images.

### NICD puncta quantification

To visualize the NICD distribution, the entire wild type and *cup*^*01355*^ stage 8 egg chambers were imaged in Zeiss LSM 710 confocal microscope or Zeiss Axio observer 7 with Apotome.2 module. z sections were acquired at regular intervals of 40X magnification. For quantifying the NICD puncta of anterior follicle cells, z-sections encompassing the anterior follicle cells were extracted. The apical membrane marked by NICD proteins was used as a reference to delineate follicle cells from the nurse cells. The z images at regular intervals of 0.68 μm were used for counting the NICD puncta.

The particles were counted using the Image J software. The AFCs which can take future BCs’ fate without apical nurse cell-follicle cell junction, were outlined for every image. A threshold value was selected on the basis that each NICD puncta were detected as an individual spot, and the background was excluded. The particles were automatically counted by the <Analyze Particles> option. The average radius of NICD particles was measured and found to be within 0.6 μm. The particle size value range was set from 0.2–1.2 μm^2,^ and circularity was set from 0.5–1. The particles were counted for 9 egg chambers each of control and *cup*^*01355*^ and the total count of NICD particles was plotted with statistical tests.

### NECD puncta quantification

To visualize the NECD distribution, the entire wild type and *cup*^*01355*^ stage 8 egg chambers were imaged in Zeiss LSM 710 confocal microscope or Zeiss Axio observer 7 with Apotome.2 module. Z sections were acquired at regular intervals of 40X magnification. For quantifying the NECD puncta in the nurse cell cytoplasm, z-sections encompassing the nurse cells were extracted. The z-section images at regular intervals of 0.68 μm were used for counting the puncta to avoid overlapping puncta amongst the z-planes.

The particles were counted using the Image J software. The nurse cell area, excluding the follicle cells and oocyte, was outlined for every image. A threshold value was selected on the basis that each NECD puncta were detected as an individual spot, and the background was excluded. The particles were automatically counted by the <Analyze Particles> option. The average radius of NECD particles was measured and found to be within 0.6 μm. The particle size value range was set from 0.2–1.2 μm^2,^ and circularity was set from 0.5–1. The particles were counted for 9 egg chambers each of control and *cup*^*01355*^ and the total count of NECD particles for each plane was plotted with statistical tests.

### *upd*-lacZ intensity quantification

The *upd*-lacZ fly stock was a kind gift from Prof. Henry Sun. *upd*-lacZ consists of a regulatory sequence of the Upd gene driving the expression of lacz, which reflects the transcriptional status of Upd locus.

For determining the lacZ protein levels, immunostaining was performed using a primary antibody against the β-Gal protein. The polar cells at the anterior end of egg chambers were imaged at 40X taking z sections at regular intervals keeping equal exposure for experiment and control. All the stacks were merged to obtain a 2-dimensional maximum intensity projection (MIP) image. The polar cells were outlined in the MIP image, and mean lacZ intensity was obtained using the Zen 2012 software. Images were acquired in Zeiss Axio observer 7 with Apotome.2 module.

### Statistical test

Two-tailed t-test of unequal variance in Excel and GraphPad Prism 6.0 were used to determine the statistical significance. The standard Error of the Mean value was used to plot the error bars. A range used for assigning the p-value is as follows: p-value <0.001 is designated as ***, p-value <0.01 is designated as **, and 0.05< p <0.01 is designated as *. n = number of egg chambers.

## Supporting information

S1 Fig Cup functions in nurse cells to regulate border cell cluster size. (**A-D**) Respective homozygous mutant egg chambers exhibit larger border cell clusters compared to wild type, Armadillo (red). The white dotted line marks the BC cluster. *** indicates a p value <0.001 (Student t-test). **(E-G)** Hetero allelic combination of *cup*^*8*^ and *cup*^*01355*^ exhibits increased border cell numbers, F-actin (magenta), DAPI (blue, grey in inset) and white arrow heads mark the border cell cluster. **(H-I)** *cup*^*01355*^ exhibit reduced *cup* transcripts, normalised to *rp49* compared to wild type. ** indicates a p value <0.01 (Student t-test). **(J-K)** Anterior Follicle Cell clones mutant for *cup*^*15*^ marked by GFP (green), Armadillo (red), DAPI (blue, grey in inset) does not alter number of BCs (white arrow head) compared to control (FRT 40A). ns represents statistically nonsignificant. **(L-O)** Increased BC number (white arrow head) is not rescued by *UASp-CupCDS*, driven by *c306-*GAL4 in *cup*^*01355*^ egg chambers, F-actin (magenta), DAPI (cyan, grey in inset). **** indicates a p value <0.0001 (Student t-test). ns represents statistically nonsignificant n represents the number of egg chambers evaluated in each panel.
(TIF)

S2 Fig Cup mutation does not affect the timing of mitotic to endoreplication switch in the developing follicle cells. **(A-H)** Expression endoreplication marker Cut in *cup*^*01355*^ early and late-stage (> stage10) egg chambers. Cut (red), F-actin (green), DAPI (blue). **(I-N’)** Phospho histone 3 (pH3) staining is observed only in early-stage egg chambers (up to stage 6) in both wild type and *cup*^*01355*^ egg chambers (yellow arrow head marks the pH3). The presence of FasII indicates it to be early egg chamber. pH3 staining is not observed in stage 8/9 egg chambers of wild type and *cup*^*01355*^ egg chambers indicating no cell proliferation post stage 7. **(O-Q)** Total number of Follicle Cells are unchanged in stage 8 egg chambers of *cup*^*01355*^ compared to wild type, DAPI (grey). ns represents statistically nonsignificant n represents the number of egg chambers evaluated in each panel. SEM represents the error bars.
(TIF)

S3 Fig Cup mutation does not affect polar cell specification nor the transcription of Upd. **(A-B)** Number of polar cells is same in wild type and *cup*^*01355*^ stage 8 egg chamber indicated by FasIII staining (red). F-actin (green), DAPI (blue). The yellow arrow head marks the polar cells. **(C-J’)** The number of polar cells is the same in the early stages of oogenesis (stage2-7) in wild type and *cup*^*01355*^ egg chambers indicated by FasIII staining (red), and DAPI (blue). **(K-M)** *upd-*lacZ intensity of polar cells is similar in stage 8 for wild type and *cup*^*01355*^ egg chambers. lacZ (red), F-actin (green), DAPI (blue), and yellow arrow head indicate the polar cells. **(N-V)** *upd-*lacZ intensity of polar cells is not changed in early-stage (2–7) *cup*^*01355*^ egg chambers as compared to wild type. lacZ (red), DAPI (blue), and yellow arrow head indicates polar cells. Error bars represent SEM, ns represents statistically nonsignificant (student t-test).
(TIF)

S4 Fig Germline cup regulates the Notch signalling in AFCs. **(A-C)** Stage 8 egg chambers exhibit reduced number of NICD puncta (yellow arrow head) in follicle cells in hetero allelic combination of *cup*^*8*^ and *cup*^*01355*^ genetic background. **(D-G)** NRE-eGFP intensity is rescued by *UASp-CupCDS*, driven by *nos* GAL4*-VP16* in *cup*^*01355*^ egg chambers NRE-eGFP (green), DAPI (blue, grey in inset). **(H-K)** NRE-eGFP intensity is not rescued when *UASp-CupCDS* is driven by *c306-*GAL4 in the *cup*^*01355*^ egg chambers, NRE-eGFP (green), DAPI (blue, grey in inset). Error bars represent SEM, ns represents statistically nonsignificant (student t-test). **** and *** indicates a p value <0.0001 and <0.001 respectively (Student t-test). n represents the number of egg chambers evaluated in each panel.
(TIF)

S5 Fig Delta trafficking is perturbed in the heteroallelic combination of *cup*^*8*^ and *cup*^*01355*^ egg chambers. **(A-C)** Delta stained in hetero allelic *cup*^*8*^ and *cup*^*01355*^ egg chambers exhibit more cytoplasmic puncta in nurse cells as compared to wild type (yellow arrow heads), delta (black), DAPI (cyan). Error bars represent SEM, **** indicates a p value <0.0001 (Student t-test). **(D)** Over expression of actin and tubulin in nurse cells of *cup*^*01355*^ egg, chambers do not rescue border cell numbers. ns represents statistically nonsignificant (student t-test). n represents the number of egg chambers evaluated in each panel.
(TIF)

S6 Fig Overexpression of Rab11CA rescues the actin filaments. **(A-G)** Stage 10 egg chambers of indicated genotypes stained with EYA (magenta), DAPI (blue, grey in inset), and YFP (green), yellow arrow heads mark the border cell cluster. **(H-K)** Rescue of F-Actin when Rab11^CA^ is overexpressed in the nurse cells of *cup*^*01355*^ egg chambers compared to that observed alone in the *cup*^*01355*^ egg chambers. Yellow arrow heads indicate the actin fibres, F-actin (grey, green in inset), DAPI (red). **** indicates a p value <0.0001 (Student t-test). Error bars represent SEM, n represents the number of egg chambers evaluated.
(TIF)

S1 Table List of 14 candidate genes shortlisted from the flybase that are expressed in the nurse cells and have female sterile phenotype. (TIFF)
