# Supplementary figures and images for: Germline protein, Cup, non-cell autonomously limits migratory cell fate in Drosophila oogenesis
Source: PLoS Genet. 2023 Feb 15;19(2):e1010631. doi: 10.1371/journal.pgen.1010631 (PMC9974129; doi:10.1371/journal.pgen.1010631)

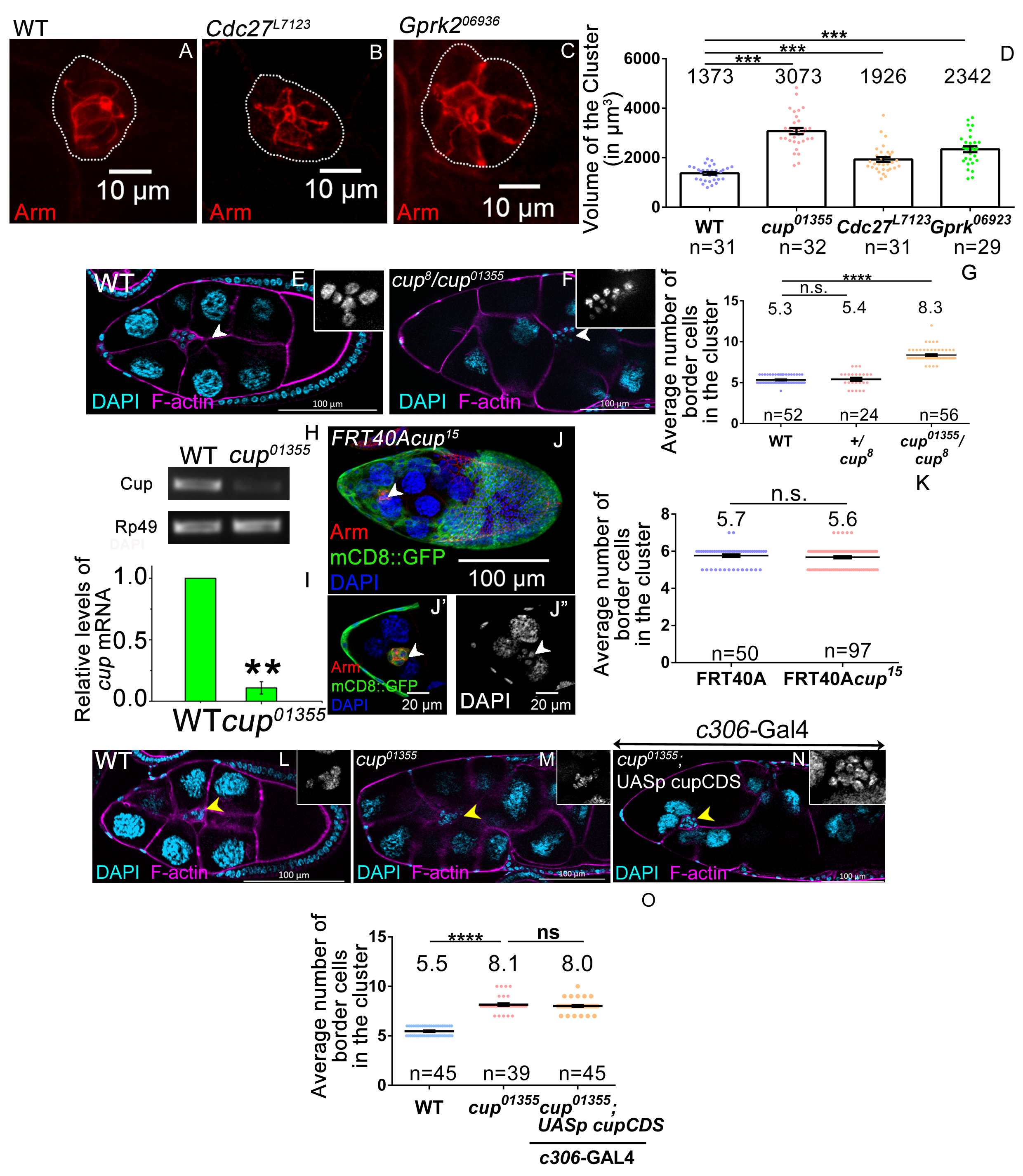

Supplement: S1 Fig — (A-D) Respective homozygous mutant egg chambers exhibit larger border cell clusters compared to wild type, Armadillo (red). The white dotted line marks the BC cluster. *** indicates a p value <0.001 (Student t-test). (E-G) Hetero allelic combination of cup8 and cup01355 exhibits increased border cell numbers, F-actin (magenta), DAPI (blue, grey in inset) and white arrow heads mark the border cell cluster. (H-I) cup01355 exhibit reduced cup transcripts, normalised to rp49 compared to wild type. ** indicates a p value <0.01 (Student t-test). (J-K) Anterior Follicle Cell clones mutant for cup15 marked by GFP (green), Armadillo (red), DAPI (blue, grey in inset) does not alter number of BCs (white arrow head) compared to control (FRT 40A). ns represents statistically nonsignificant. (L-O) Increased BC number (white arrow head) is not rescued by UASp-CupCDS, driven by c306-GAL4 in cup01355 egg chambers, F-actin (magenta), DAPI (cyan, grey in inset). **** indicates a p value <0.0001 (Student t-test). ns represents statistically nonsignificant n represents the number of egg chambers evaluated in each panel. (TIF) [file pgen.1010631.s001.tif]

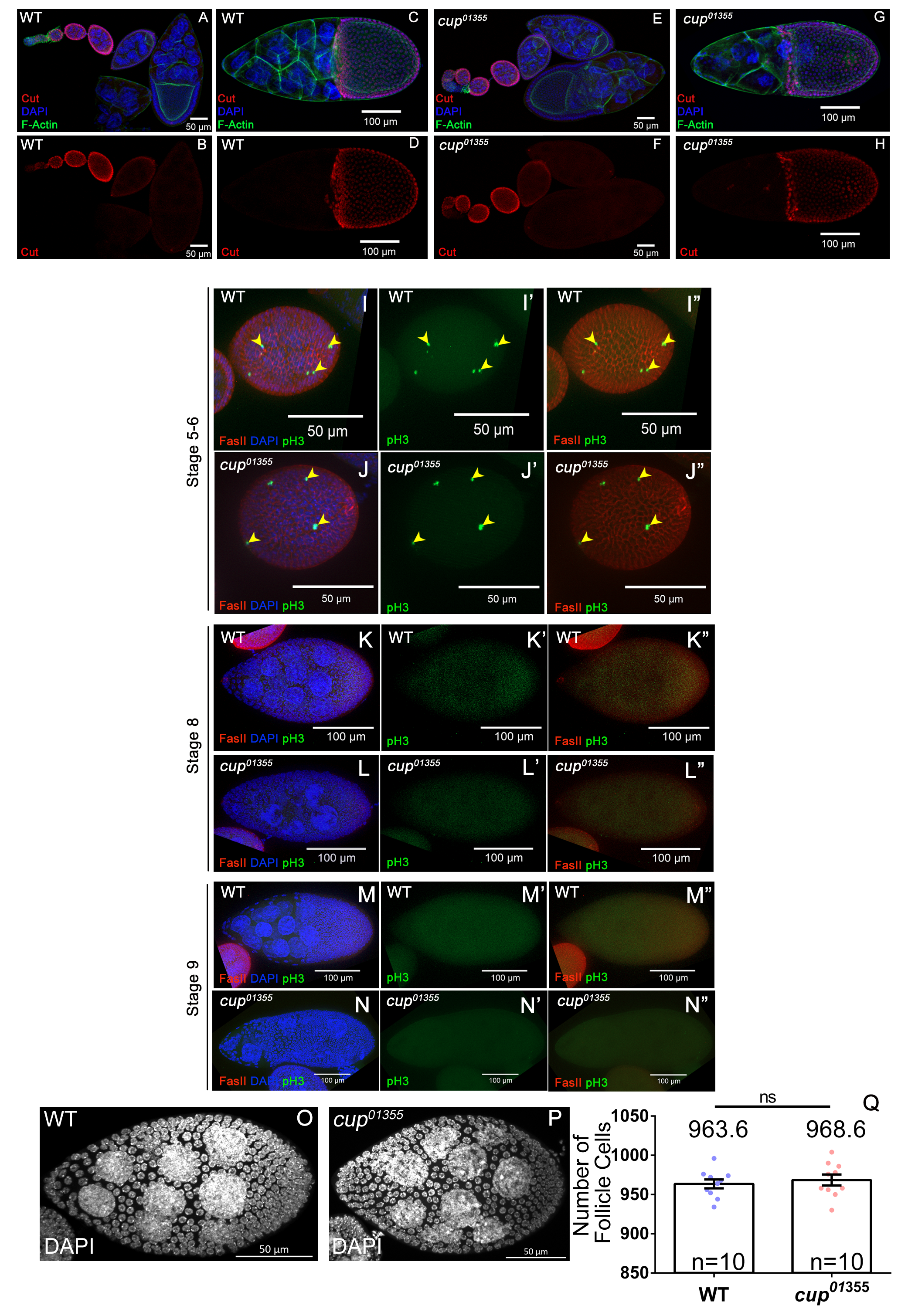

Supplement: S2 Fig — (A-H) Expression endoreplication marker Cut in cup01355 early and late-stage (> stage10) egg chambers. Cut (red), F-actin (green), DAPI (blue). (I-N’) Phospho histone 3 (pH3) staining is observed only in early-stage egg chambers (up to stage 6) in both wild type and cup01355 egg chambers (yellow arrow head marks the pH3). The presence of FasII indicates it to be early egg chamber. pH3 staining is not observed in stage 8/9 egg chambers of wild type and cup01355 egg chambers indicating no cell proliferation post stage 7. (O-Q) Total number of Follicle Cells are unchanged in stage 8 egg chambers of cup01355 compared to wild type, DAPI (grey). ns represents statistically nonsignificant n represents the number of egg chambers evaluated in each panel. SEM represents the error bars. (TIF) [file pgen.1010631.s002.tif]

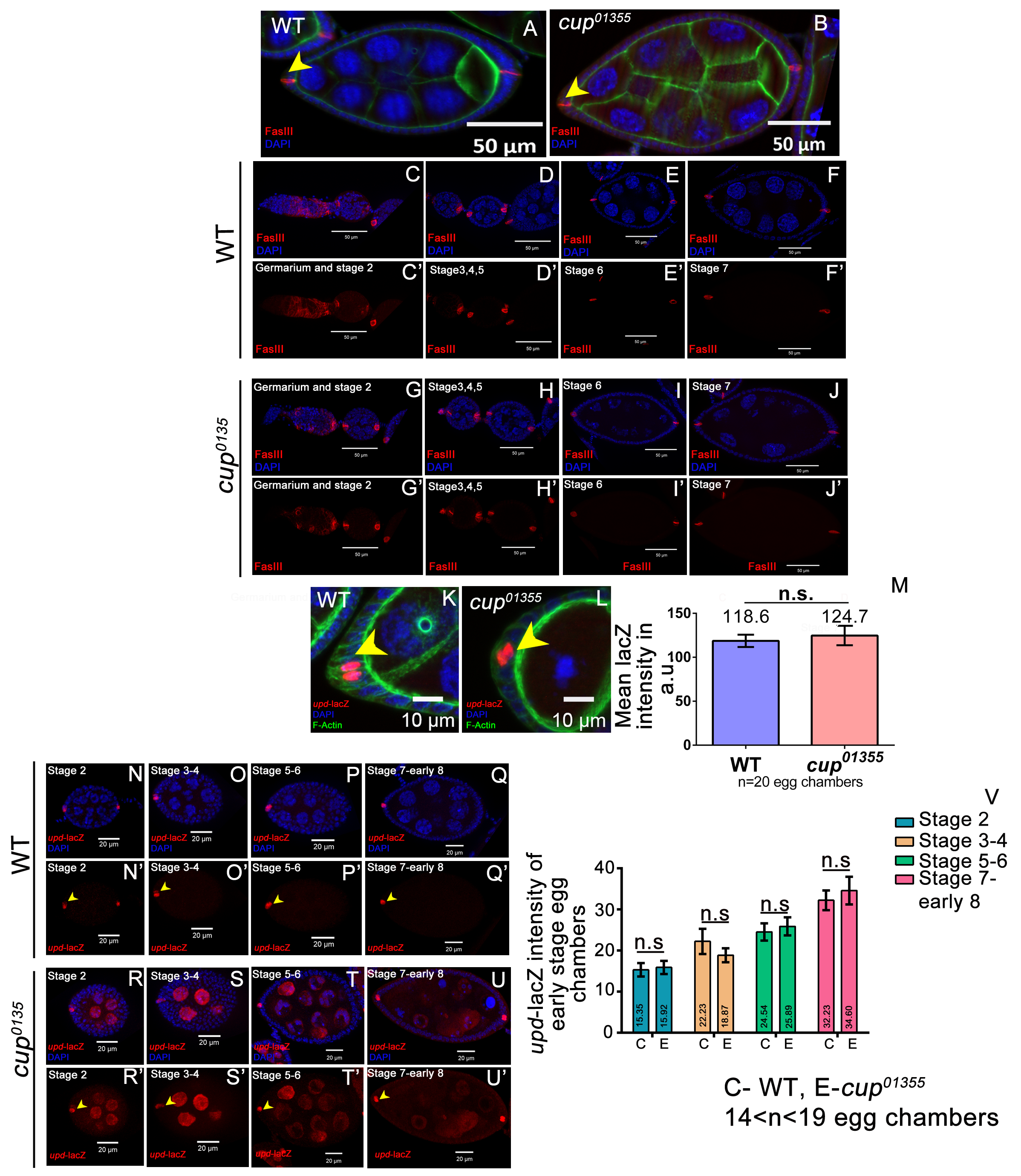

Supplement: S3 Fig — (A-B) Number of polar cells is same in wild type and cup01355 stage 8 egg chamber indicated by FasIII staining (red). F-actin (green), DAPI (blue). The yellow arrow head marks the polar cells. (C-J’) The number of polar cells is the same in the early stages of oogenesis (stage2-7) in wild type and cup01355 egg chambers indicated by FasIII staining (red), and DAPI (blue). (K-M) upd-lacZ intensity of polar cells is similar in stage 8 for wild type and cup01355 egg chambers. lacZ (red), F-actin (green), DAPI (blue), and yellow arrow head indicate the polar cells. (N-V) upd-lacZ intensity of polar cells is not changed in early-stage (2–7) cup01355 egg chambers as compared to wild type. lacZ (red), DAPI (blue), and yellow arrow head indicates polar cells. Error bars represent SEM, ns represents statistically nonsignificant (student t-test). (TIF) [file pgen.1010631.s003.tif]

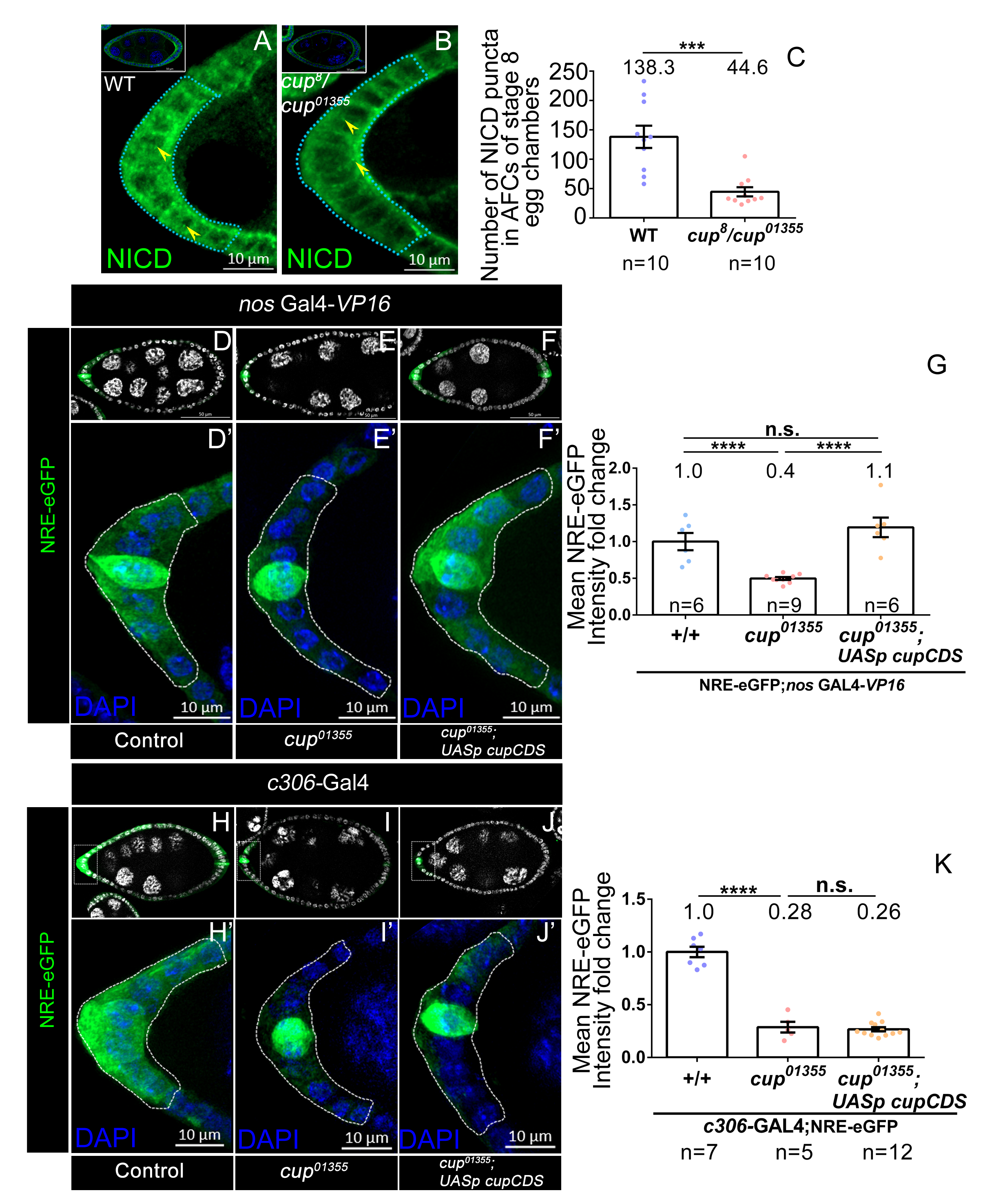

Supplement: S4 Fig — (A-C) Stage 8 egg chambers exhibit reduced number of NICD puncta (yellow arrow head) in follicle cells in hetero allelic combination of cup8 and cup01355 genetic background. (D-G) NRE-eGFP intensity is rescued by UASp-CupCDS, driven by nos GAL4-VP16 in cup01355 egg chambers NRE-eGFP (green), DAPI (blue, grey in inset). (H-K) NRE-eGFP intensity is not rescued when UASp-CupCDS is driven by c306-GAL4 in the cup01355 egg chambers, NRE-eGFP (green), DAPI (blue, grey in inset). Error bars represent SEM, ns represents statistically nonsignificant (student t-test). **** and *** indicates a p value <0.0001 and <0.001 respectively (Student t-test). n represents the number of egg chambers evaluated in each panel. (TIF) [file pgen.1010631.s004.tif]

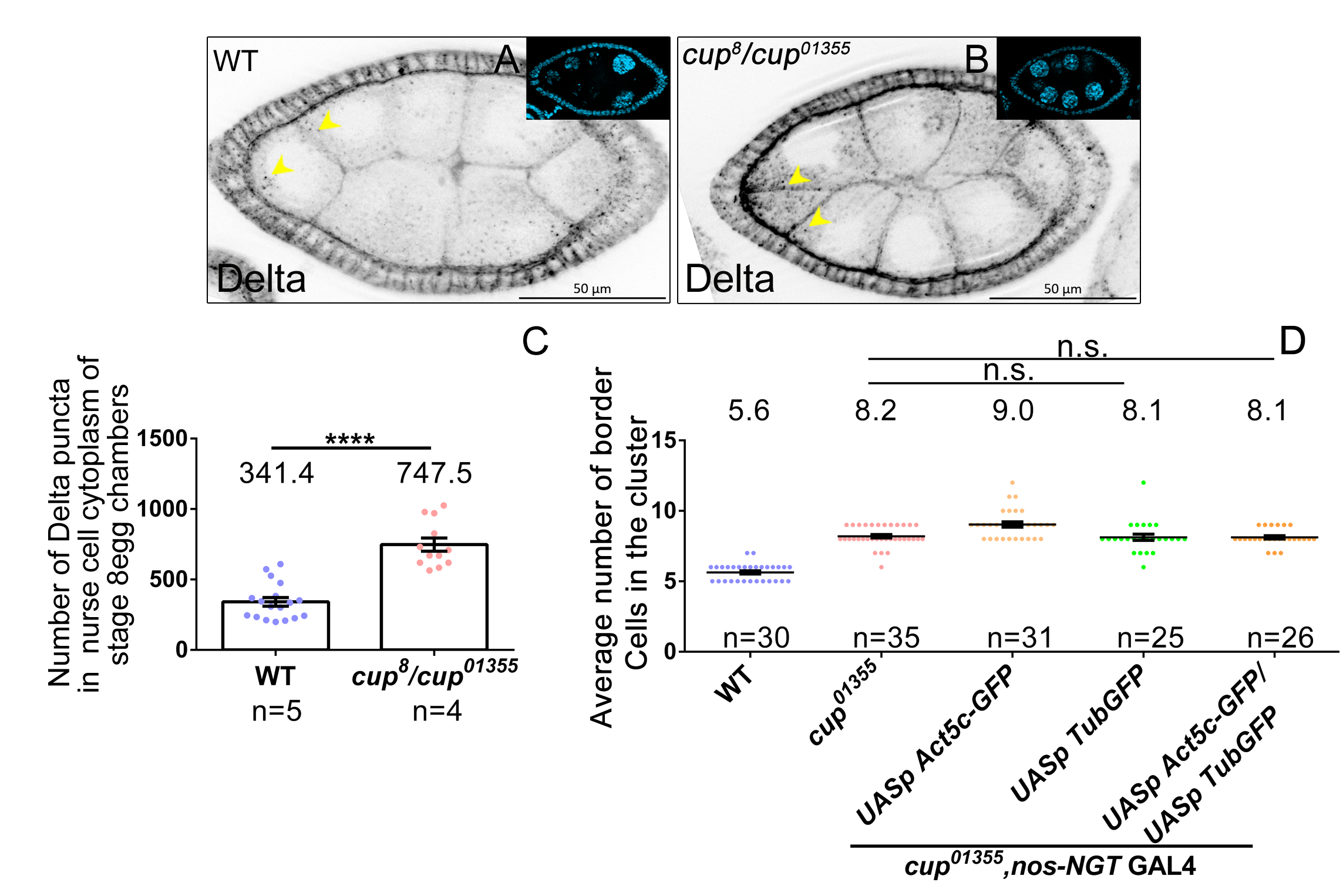

Supplement: S5 Fig — (A-C) Delta stained in hetero allelic cup8 and cup01355 egg chambers exhibit more cytoplasmic puncta in nurse cells as compared to wild type (yellow arrow heads), delta (black), DAPI (cyan). Error bars represent SEM, **** indicates a p value <0.0001 (Student t-test). (D) Over expression of actin and tubulin in nurse cells of cup01355 egg, chambers do not rescue border cell numbers. ns represents statistically nonsignificant (student t-test). n represents the number of egg chambers evaluated in each panel. (TIF) [file pgen.1010631.s005.tif]

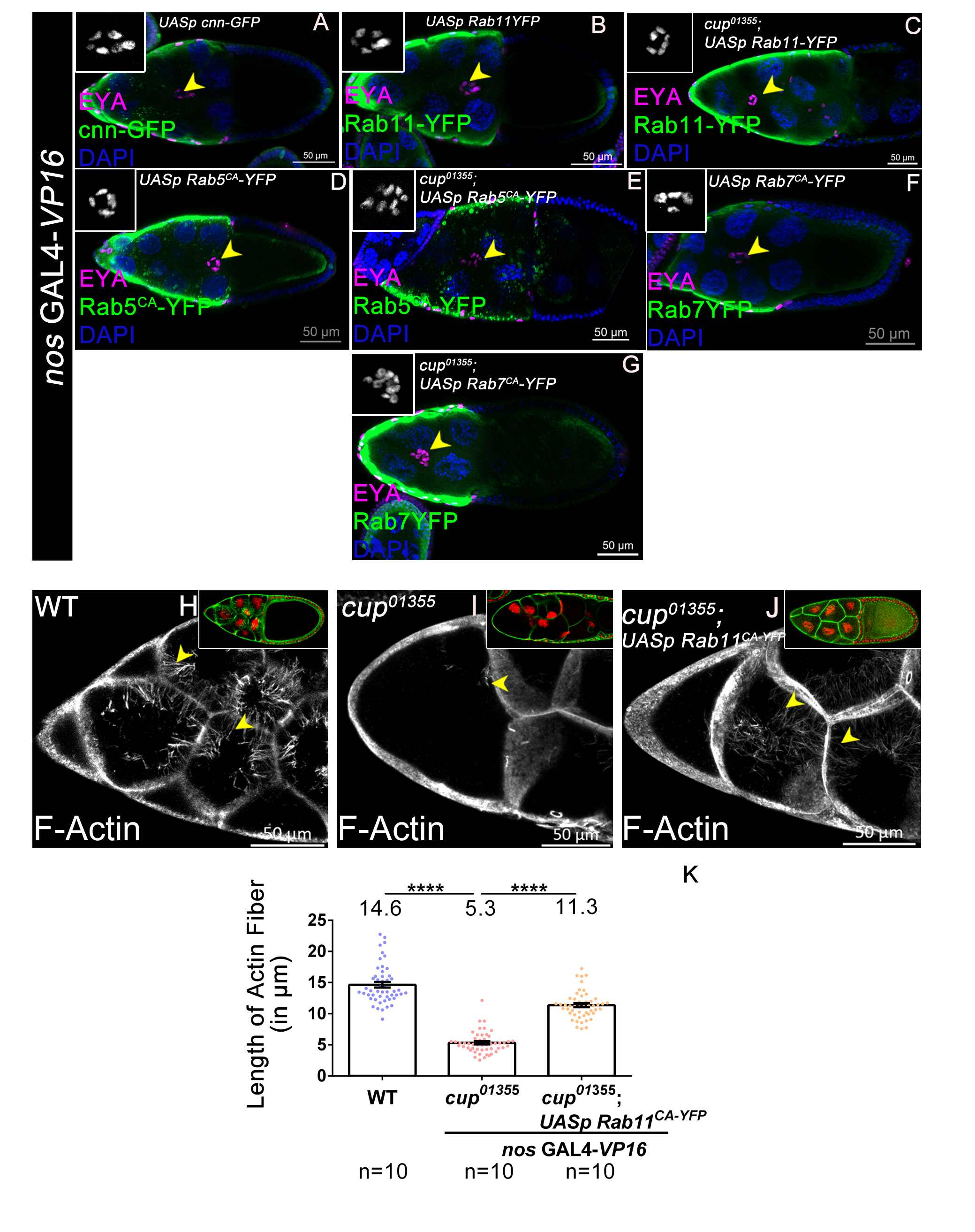

Supplement: S6 Fig — (A-G) Stage 10 egg chambers of indicated genotypes stained with EYA (magenta), DAPI (blue, grey in inset), and YFP (green), yellow arrow heads mark the border cell cluster. (H-K) Rescue of F-Actin when Rab11CA is overexpressed in the nurse cells of cup01355 egg chambers compared to that observed alone in the cup01355 egg chambers. Yellow arrow heads indicate the actin fibres, F-actin (grey, green in inset), DAPI (red). **** indicates a p value <0.0001 (Student t-test). Error bars represent SEM, n represents the number of egg chambers evaluated. (TIF) [file pgen.1010631.s006.tif]

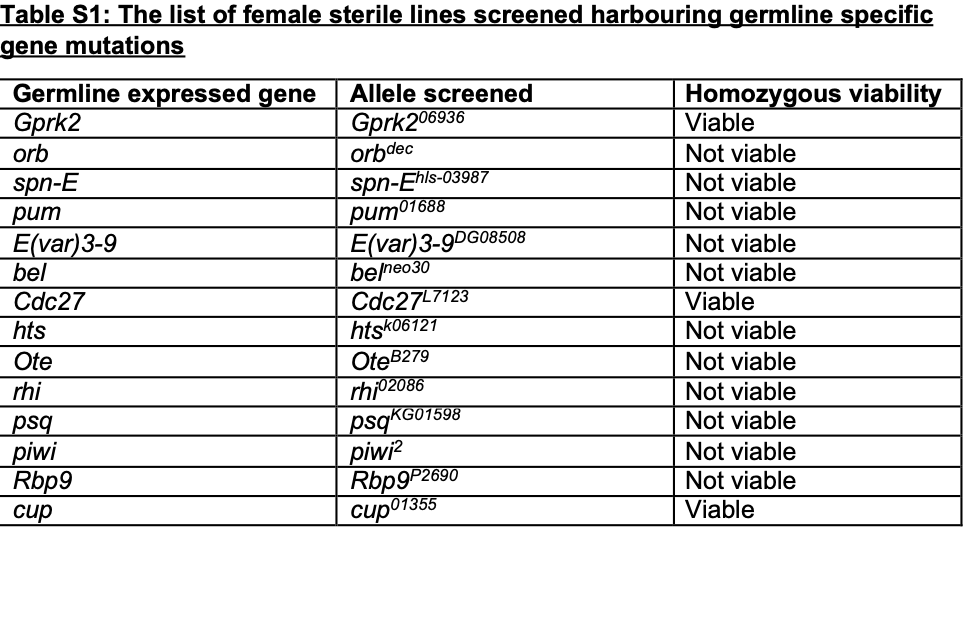

Supplement: S1 Table — (TIFF) [file pgen.1010631.s007.tiff]
